# Solvent-Free Method for Defect Reduction and Improved Performance of p-i-n Vapor-Deposited Perovskite Solar Cells

**DOI:** 10.1021/acsenergylett.2c00865

**Published:** 2022-05-09

**Authors:** Kilian B. Lohmann, Silvia G. Motti, Robert D. J. Oliver, Alexandra J. Ramadan, Harry C. Sansom, Qimu Yuan, Karim A. Elmestekawy, Jay B. Patel, James M. Ball, Laura M. Herz, Henry J. Snaith, Michael B. Johnston

**Affiliations:** † Department of Physics, Clarendon Laboratory, University of Oxford, Parks Road, Oxford OX1 3PU, United Kingdom; ‡ Institute for Advanced Study, Technical University of Munich, Lichtenbergstrasse 2a, D-85748 Garching, Germany

## Abstract

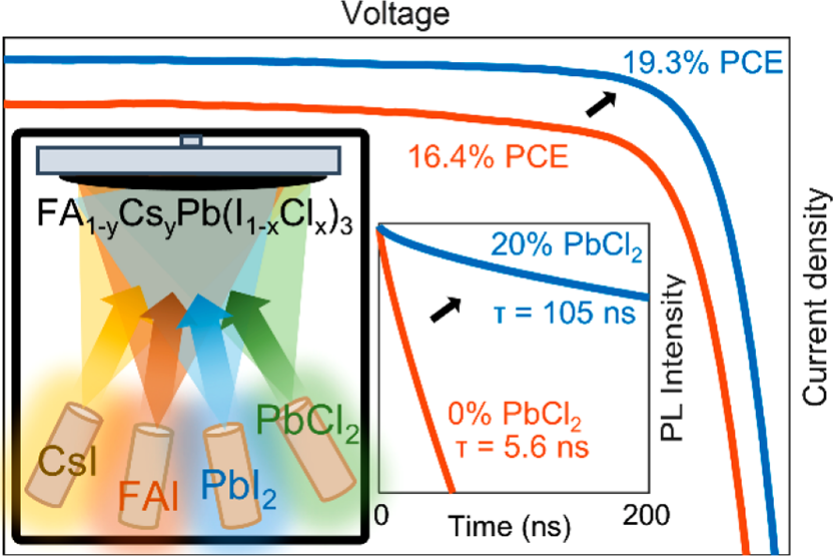

As perovskite-based photovoltaics near commercialization, it is imperative to develop industrial-scale defect-passivation techniques. Vapor deposition is a solvent-free fabrication technique that is widely implemented in industry and can be used to fabricate metal-halide perovskite thin films. We demonstrate markably improved growth and optoelectronic properties for vapor-deposited [CH(NH_2_)_2_]_0.83_Cs_0.17_PbI_3_ perovskite solar cells by partially substituting PbI_2_ for PbCl_2_ as the inorganic precursor. We find the partial substitution of PbI_2_ for PbCl_2_ enhances photoluminescence lifetimes from 5.6 ns to over 100 ns, photoluminescence quantum yields by more than an order of magnitude, and charge-carrier mobility from 46 cm^2^/(V s) to 56 cm^2^/(V s). This results in improved solar-cell power conversion efficiency, from 16.4% to 19.3% for the devices employing perovskite films deposited with 20% substitution of PbI_2_ for PbCl_2_. Our method presents a scalable, dry, and solvent-free route to reducing nonradiative recombination centers and hence improving the performance of vapor-deposited metal-halide perovskite solar cells.

Organic–inorganic metal halide perovskites (MHP) are a novel class of semiconductors that have leapt to the forefront of photovoltaic (PV) research following the unprecedented growth in their power conversion efficiency (PCE),^1,2^ which has now reached 25.7% in single junction devices.^3^ In particular, this material has garnered interest because of its band gap tunability^4−8^ and compatibility with a range of manufacturing approaches,^9^ making it an ideal candidate for tandem applications in all-perovskite configurations^10,11^ or in combination with established silicon technologies.^12−14^ With perovskite-on-silicon tandems recently reaching 29.8% certified PCE,^3^ MHP solar cell technology is poised to deliver the next major advancement in efficiency for the photovoltaics industry.

The remarkable progress of MHP solar cells in the past few years has in a large part resulted from the development and improvement of thin-film crystallization and passivation techniques, aimed at reducing the defect density in the semiconducting perovskite layers and minimizing nonradiative recombination at defects within the bulk of the crystal, at its surfaces, and at interfaces with other materials. Improving the thin-film growth has been enabled by controlling “precursor-phases” during the thin-film deposition and crystallization, typically by employing mixed-solvent systems^15,16^ and tuning the solvent lead-halide complexation.^17,18^ In particular, the introduction of an “antisolvent quenching” step resulted in a step improvement in thin-film quality and reproducibility.^15,16^ The most successful passivation routes have been through a combination of molecular passivation,^19^ typically with primary amines or ammonium molecules,^20^ or via the creation of extremely thin 2D layered-phase perovskites on top of, or mixed within, the 3D perovskite film.^21^ Furthermore, the addition of a small excess of chloride salt to the starting solution has been shown to enhance crystallization of the perovskite thin films leading to improved optoelectronic properties.^13,22,23^ However, it is not clear if this Cl addition is simply a component that aids the crystallization, or if the residual presence of Cl is playing an active role in the crystallized films. Notably, significant Cl miscibility has been demonstrated in state-of-the-art FA_0.75_Cs_0.25_Pb(I_0.8_Br_0.2_)_3_ compositions, leading to significant improvements in perovskite films for tandem applications.^13^

A significant challenge to the wide-scale adoption of MHP technology is developing an industrially scalable and compatible manufacturing technique. Vacuum deposition, where precursors are heated under vacuum to sublimate and condense onto the desired substrate, is an ideal candidate, as it allows for conformal coating of large substrates,^24^ fine control over the thickness of the perovskite layer,^25^ and deposition in a highly controlled atmosphere. Therefore, while solution processing routes for MHPs may be more cost-effective at the small scale (particularly in terms of capital investment), at industrial scale vapor deposition offers significant cost advantages in terms of device yield as well as avoiding economic and environmental costs associated with solvent procurement and disposal.^26^ Furthermore, vapor deposition has particular potential for the fabrication of multilayer devices, such as tandem and multijunction solar cells,^27,28^ where layers of different materials must be deposited sequentially and precise control over film thickness is critical. Indeed, vacuum deposition readily allows multiple layers to be deposited on top of each other without damaging the previous layers and without solvent orthogonality constraints.^26^ Having already been used in other commercial optoelectronic devices such as OLED displays^9,27^ and copper indium gallium (di)selenide thin-film PV modules,^29^ vacuum deposition is a proven technology readily applicable to upscale MHP solar cell production.

However, despite being highly compatible with large-scale manufacturing, the cost of research scale equipment for vapor deposition is significantly higher than the cost of a spin-coater. This has led to a vast asymmetry in the amount of research effort undertaken on MHP vapor deposition, in comparison to solution processing. Nevertheless, high-efficiency vacuum processed MHP solar cells (PCE 21.3%) have been demonstrated through sequential sublimation of the precursor materials to form FA_0.95_Cs_0.05_PbI_3_,^30^ while large-area (21 cm^2^) MAPbI_3_ mini-modules deposited through precursor coevaporation have reached a respectable 18.1% PCE.^24^ Significant progress has also been achieved replicating state-of-the-art mixed-cation and mixed-halide MHP compositions using up to four deposition sources^31−36^ and the stabilization of FAPbI_3_ through vacuum deposition.^33,35^ However, high-efficiency vapor-deposited devices often still rely on the use of MAI as a precursor,^33,35,36^ which can thermally degrade at temperatures as low as 80 °C,^37,38^ and has been shown to have inconsistent sublimation behavior,^39−42^ highlighting the importance of developing MA-free MHP formulations.^32^ While a lot of the advancements in vapor deposited MHPs have benefited from the learning gained with the solution processed perovskites, most of the methodologies for thin-film growth control and passivation are not translatable to dry solvent-free processing.

Herein, we investigate if the addition of controllable amounts of Cl-salts to the growth of MA-free lead-triiodide perovskite thin films can influence the crystallization and eventual optoelectronic quality of the vapor deposited thin films. We discover that substituting varying amounts of PbI_2_ for PbCl_2_ in a coevaporated mixed-cation triiodide system, namely, FA_0.83_Cs_0.17_PbI_3_, results in a profoundly positive impact upon both optoelectronic quality and subsequent solar-cell performance. We then discuss the origin of these improvements and find evidence that Cl is retained in the final perovskite films.

We fabricated a series of FA_1–*y*_Cs_*y*_Pb(I_1–*x*_Cl_*x*_)_3_ perovskite thin films via simultaneous codeposition of four precursors (FAI, CsI, PbI_2_, PbCl_2_), each in its own independently controlled vacuum furnace in a custom-built vacuum deposition system. We started with the neat iodide system of FA_0.83_Cs_0.17_PbI_3_ using a thickness of 500 nm. Then, for each growth run, the PbI_2_ precursor was progressively substituted by PbCl_2_, such that the total dose of Pb and halide atoms remained the same for all depositions. For example, a 30% PbCl_2_ substitution film was formed from 70% PbI_2_ and 30% PbCl_2_ by molar amount. FAI and CsI deposition rates were kept the same for all films. After deposition, the films were annealed in an N_2_ glovebox, at 135 °C for 30 min.^32^ The compositions investigated were 0% (neat I), 10%, 20%, 30%, and 40% PbCl_2_ substitution. Because additional iodine is introduced by the A-site precursors (CsI, FAI), the actual dose of Cl sublimated as a percentage of total halide is given by [Cl]/([Cl] + [I]) = 0%, 7%, 13%, 20%, and 27% (rounded to the nearest integer percentage). As discussed later, there is uncertainly related to where and how much Cl ends up in the final film and how it affects the FA/Cs ratio in the perovskite phase. Thus, we will henceforth use the former nomenclature of the PbCl_2_ substitution percentage to denote the films. In addition, the films were fabricated with a slight lead halide excess, even for the neat I sample, in accordance to state-of-the-art practices for vapor deposited MHP.^30,32,43^

The substitution of PbI_2_ with PbCl_2_ in vapor codeposition has a dramatic effect on the optoelectronic properties of the resulting perovskite films. We start by comparing a FA_1–*y*_Cs_*y*_Pb(I_1–*x*_Cl_*x*_)_3_ film of intermediate PbCl_2_ substitution (20%) with a reference film of neat iodide FA_0.83_Cs_0.17_PbI_3_. Both films formed high-quality α-phase perovskite polycrystalline films, as can be seen by the X-ray diffraction (XRD) patterns displayed in Figure 1a. The optical absorption data in Figure 1b attest to high optical quality and smoothness of both films, with a sharp onset of absorption at the bandgap energy and no significant artifact from optical scattering below the bandgap energy. Significantly, the PbCl_2_-substituted film shows a ∼65% increase in absorption coefficient combined with a slight blueshift and sharpening of the absorption edge (quantified by Elliott fitting of the absorption spectra shown, Table S6). Moreover, photocurrent spectra displayed in Figure 1c reveals a very low Urbach energy (*E*_U_) of 10.7 meV, which is consistent with low electronic disorder and hence the high electronic quality of the films. To the best of our knowledge, this is the lowest value of *E*_U_ reported for this type of MHP.

**Figure 1:**
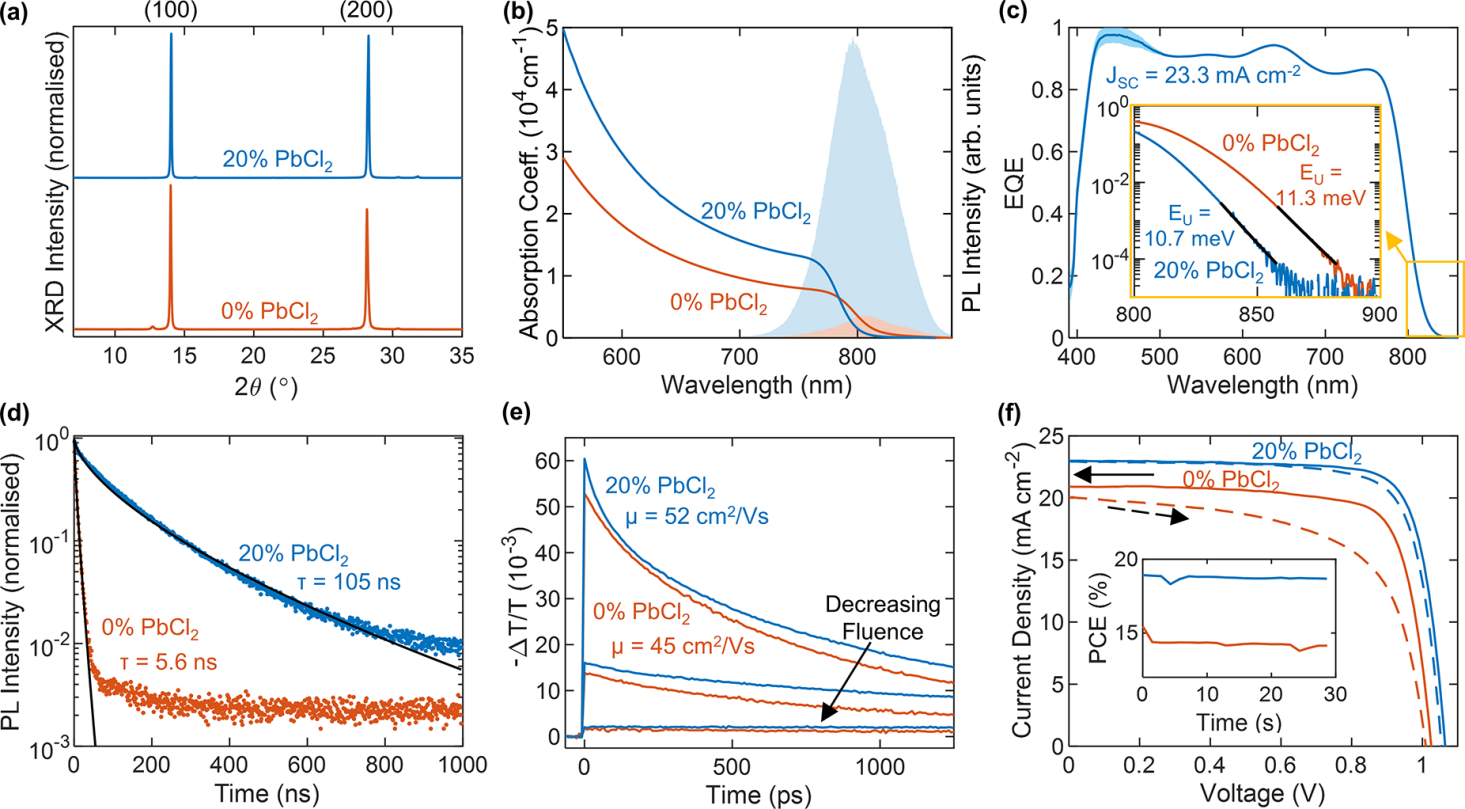
PbCl_2_ substitution improves device performance. (a) X-ray diffraction (XRD) patterns of FA_1–*y*_Cs_*y*_Pb(I_1–*x*_Cl_*x*_)_3_ full photovoltaic devices after testing, grown with (blue line) and without (red line) PbI_2_ substituted for PbCl_2_, as denoted in the figure. The XRD patterns were acquired with a Cu–K_α_ 1.54 Å X-ray source, corrected for specimen displacement, and normalized. (b) Absorption coefficient of bare thin films of the aforementioned composition on z-cut quartz. The shaded region shows the unnormalized PL after photoexcitation at 470 nm. (c) External quantum efficiency (EQE) of a device with 20% PbCl_2_ and the corresponding integrated short-circuit current (*J*_SC_). The inset shows the absorption edge from the EQE and the Urbach tail fit (black line). (d) Time-resolved photoluminescence (PL) traces for thin films of the aforementioned bare films on quartz, after photoexcitation by a 1 MHz pulsed 470 nm laser at a fluence of 20 μJ/cm^2^. The black line shows a stretched exponential fit. (e) Optical-pump THz-probe (OPTP) photoconductivity transients of the aforementioned bare films on quartz after photoexcitation at 400 nm as a function of fluence (1.0, 10, 42 μJ/cm^2^). (f) Current–voltage (*J*–*V*) measurements of the aforementioned photovoltaic devices under AM1.5 illumination, as measured under reverse bias (solid line) and forward bias (dashed line). The inset shows the power conversion efficiency (PCE) measured at the max power point under continuous illumination over 30 s.

However, the most dramatic effects of PbCl_2_ substitution on vapor-deposited FA_1–*y*_Cs_*y*_Pb(I_1–*x*_Cl_*x*_)_3_ films is a resulting order-of-magnitude increase in both the photoluminescence (PL) lifetime and photoluminescence quantum yield (PLQY). Figure 1d shows the time dynamics of PL for films with and without PbCl_2_ substitution as a function of time after excitation by a 470 nm-wavelength laser pulse. The short 5.6 ns lifetime of the reference neat-iodide FA_0.83_Cs_0.17_PbI_3_ film is typical of vapor-deposited MHP films, even those used in high-efficiency solar cells.^43−45^ In contrast, the PL lifetime of the film codeposited with PbCl_2_ remarkably increased by 18 times to 105 ns. To confirm this result we performed PLQY measurements. The PLQY measured under 532 nm laser excitation at an intensity of 0.5-Sun equivalent increased by 8 times to 0.16% for the PbCl_2_ codeposited film compared with 0.02% for the neat-iodide reference. The results of these experiments are significant as they quantify defect-mediated nonradiative recombination of photogenerated charge carriers in as-grown semiconductor films^46^ and hence predict their ultimate performance in solar cell devices.

In parallel, the mobility of charge carriers is a trusted figure of merit for the performance of semiconductors and is dependent on band structure and a range of charge-carrier scattering processes.^47^ The fundamental limit to the intrinsic charge-carrier mobility (drift velocity gained of a charge carrier per unit applied electric field) for this type of MHP at room temperature is limited by phonon scattering on the order of 100 cm^2^/(V s);^47,48^ hence, the charge-carrier mobility in the materials we measure here is likely to be limited by defect scattering. Any further reduction in mobility below this level is indicative of scattering from crystal defects. We probed the intrinsic charge-carrier mobility in our FA_1–*y*_Cs_*y*_Pb(I_1–*x*_Cl_*x*_)_3_ perovskite films with optical-pump THz-probe (OPTP) spectroscopy^49^ to assess how PbCl_2_ codeposition affected the electronic properties of the films. The results of these experiments are shown in Figure 1e, where it can be seen that our reference (neat iodide) FA_0.83_Cs_0.17_PbI_3_ films were already of excellent electronic quality, possessing an exceptionally high mobility of 45 cm^2^/(V s) (averaged over electrons and holes). Significantly for the film codeposited with PbCl_2_, there is an even further (15%) increase in mobility to 52 cm^2^/(V s). The increase in mobility for the PbCl_2_ codeposition film is consistent with a reduction of the defect density on the length-scale relevant to the charge-carrier scattering. Notably, these mobility values are much larger than those reported previously for other thermally evaporated perovskites, which have been reported at 13.0 cm^2^/(V s) for MAPbI_3_,^43^ 33 cm^2^/(V s) for MAPbI_3–*x*_Cl_*x*_,^49^ and 26 cm^2^/(V s) for FAPbI_3_.^50^ Thus, we find that PbCl_2_ codeposition leads to significantly reduced nonradiative recombination in FA_1–*y*_Cs_*y*_Pb(I_1–*x*_Cl_*x*_)_3_ films, which points to a reduction in crystal defect density. We will discuss possible mechanisms for the lower defect density later.

The vastly improved optical and electronic properties of our PbCl_2_ codeposited FA_1–*y*_Cs_*y*_Pb(I_1–*x*_Cl_*x*_)_3_ indicates that it is a promising material to develop highly efficient thin-film solar cells. To test this hypothesis, we fabricated 0.25 cm^2^ and 1 cm^2^ solar cells with the following positive-intrinsic-negative (p-i-n) architecture: indium-doped tin oxide (ITO) as the bottom contact, followed by poly[bis(4-phenyl)(2,4,6-trimethylphenyl)amine (PTAA) as the hole transport layer (HTL), 500 nm of the perovskite FA_1–*y*_Cs_*y*_Pb(I_1–*x*_Cl_*x*_)_3_, C_60_ as the electron transport layer (ETL) with a thin bathocuproine (BCP) buffer layer, and capped by Ag as the top contact.

Figure 1f show the current as a function of voltage (*J*–*V*) for 0.25 cm^2^ devices with active layers deposited with and without PbCl_2_ and reveals a substantial increase in all *J*–*V* figures of merit for the devices with PbCl_2_. Indeed, the champion device PCE improves from 16.4% (14.1% steady-state) for the neat iodide film to 19.3% (18.7% steady state) for 20% PbCl_2_ substitution, through improvements in short-circuit current (*J*_SC_) from 20.9 mA/cm^2^ to 23.0 mA/cm^2^, open-circuit voltage (*V*_OC_) from 1.03 to 1.06 V, and fill factor (FF) from 0.76 to 0.79. These improvements are in excellent agreement with the improved electronic and optical properties we found between these materials, with the increased absorption coefficient (Figure 1d) leading to higher *J*_SC_ and reduced defect density (Figure 1b, 1e), resulting in improved *J*_SC_ and *V*_OC_. Specifically, the improved photoluminesence lifetimes we observe with the addition of PbCl_2_ would lead to increased *V*_OC_ through reduced nonradiative recombination at interfaces and improved *J*_SC_ through reduced nonradiative recombination in the bulk permitting additional charge carriers to reach the interfaces. We also find our *J*_SC_ from the *J*–*V* measurements in excellent agreement with that obtained from the measured external quantum efficiency (EQE) (Figure 1f). Thus, the improved electronic and optical properties of the PbCl_2_ vapor codeposited FA_1–*y*_Cs_*y*_Pb(I_1–*x*_Cl_*x*_)_3_ films does translate into significantly improved solar cell performance. Finally, we tested both 0.25 and 1 cm^2^ devices and found that the increased area only lowered efficiencies slightly, with the champion 1 cm^2^ device reaching 18.0% PCE (18.0% steady-state) (Figure S21).

We have established that PbCl_2_ substitution significantly improves both the optoelectronic properties of vapor-deposited FA_1–*y*_Cs_*y*_PbI_3_ films and the performance of solar cells fabricated from them. We now proceed to investigate the cause of this effect, specifically if the presence of Cl during growth simply influences crystallization or if Cl is incorporated into the perovskite crystal either at the surface or within its bulk, thus forming a mixed halide perovskite. To gain insight into these questions, we start by examining the effect of varying the flux ratio PbCl_2_ to of PbI_2_ during vapor deposition of FA_1–*y*_Cs_*y*_Pb(I_1–*x*_Cl_*x*_)_3_ films on the structural, optoelectronic, and electronic properties of FA_1–*y*_Cs_*y*_Pb(I_1–*x*_Cl_*x*_)_3_ while correlating these properties with the performance of solar cells devices. Thereby we obtain an understanding of the role of PbCl_2_ in forming high-quality semiconductor films and solar cell devices. The series of FA_1–*y*_Cs_*y*_Pb(I_1–*x*_Cl_*x*_)_3_ films and devices that we are now discussing were vapor deposited with 0% (neat I), 10%, 20%, 30%, and 40% PbCl_2_ substitution via the fabrication methods described earlier.

We begin by examining the composition and structure of the polycrystalline FA_1–*y*_Cs_*y*_Pb(I_1–*x*_Cl_*x*_)_3_ films as a function of PbCl_2_ substitution using XRD and X-ray photoemission spectroscopy (XPS). A careful look at the XRD patterns in Figure 1a reveal some very low intensity peaks are associated with PbI_2_ and CsPbCl_3_ inclusions. To highlight these weak diffraction peaks, we plot the XRD intensity on a log scale for the full series of films as a function of 2θ in Figure 2a (full traces Figure S1, lattice refinement Figure S3). The diffraction peaks at 2θ = 12.7°, 14°, and 15.8° are assigned to (001) PbI_2_ inclusions, the (100) FA_1–*y*_Cs_*y*_Pb(I_1–*x*_Cl_*x*_)_3_ bulk perovskite, and (100) CsPbCl_3_ perovskite inclusions, respectively. It can be seen that CsPbCl_3_ inclusions start forming at 20% PbCl_2_ substitution, with the intensity of the CsPbCl_3_ diffraction peak increasing monotonically with more PbCl_2_ (Figure S2). Furthermore, we find that, in small amounts, the presence of CsPbCl_3_ has a stabilizing effect on the resulting perovskite formed (as detailed in Figures S4 and S18). On the other hand, significant PbI_2_ inclusions are only present in the 0%, 30%, and 40% PbCl_2_ substituted films. We associate the initial drop in PbI_2_ excess with the overall reduction in PbI_2_ sublimated and the subsequent increase with the formation and re-evaporation of small amounts of FACl at the substrate (see section 6 of the Supporting Information for a more detailed treatment of the reaction kinetics).

**Figure 2:**
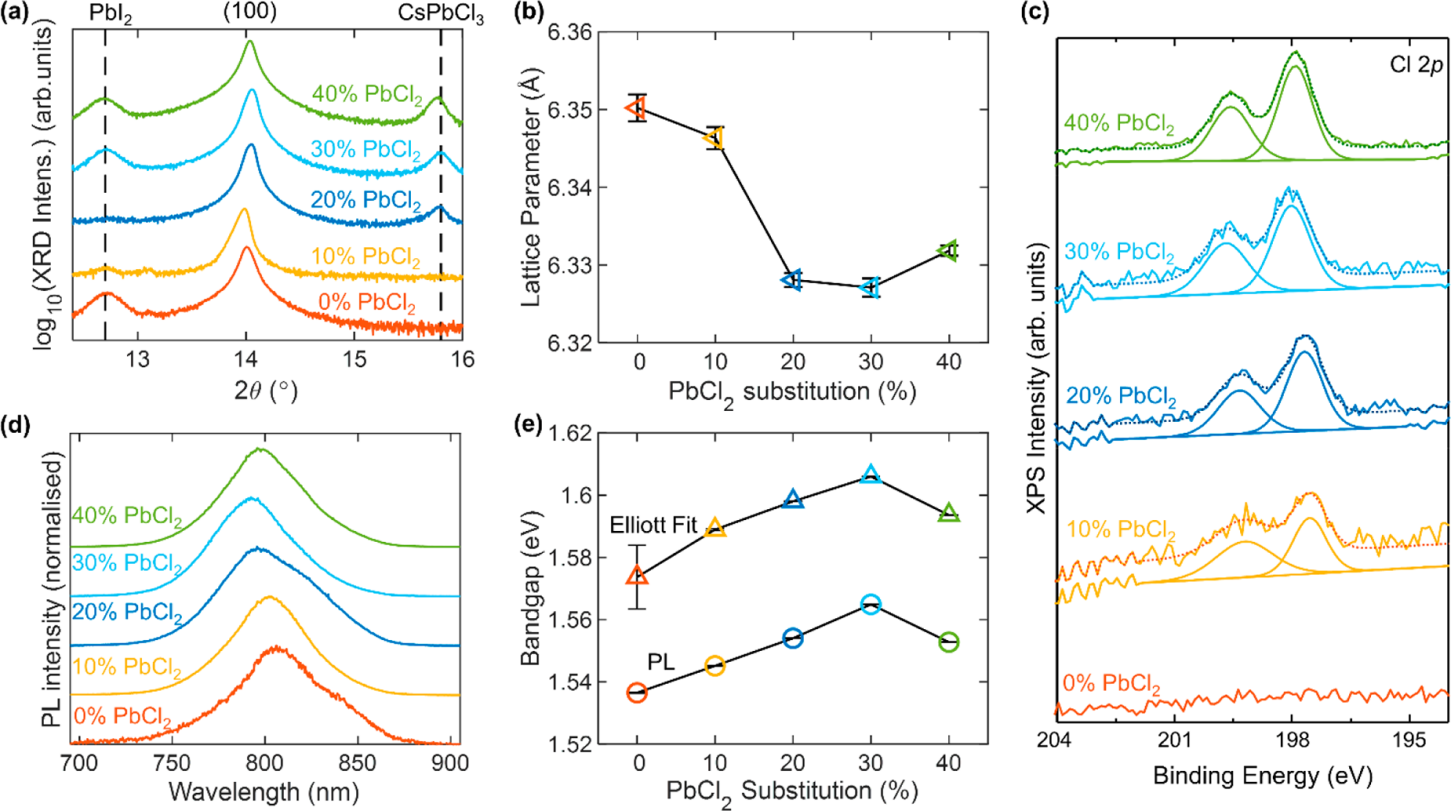
Evidence for incorporation of Cl into bulk FA_1–*y*_Cs_*y*_Pb(I_1–*x*_Cl_*x*_)_3_ perovskite. (a) X-ray diffraction patterns of FA_1–*y*_Cs_*y*_Pb(I_1–*x*_Cl_*x*_)_3_ full photovoltaic devices after testing, grown with varying amounts of PbI_2_ substituted for PbCl_2_, as depicted in the legend. The *y*-axis shows the logarithm of the measured intensity to be able to show both the large perovskite peak and the much smaller PbI_2_ and CsPbCl_3_ peaks. The XRD patterns were acquired with a Cu–K_α_ 1.54 Å X-ray source. (b) Lattice parameter obtained from the XRD traces in part a for the bulk perovskite fitted to a cubic unit cell. (c) X-ray photoemission (XPS) high-resolution spectra for thin films of the aforementioned compositions deposited on ITO/PTAA, showing the Cl 2*p* region. (d) Normalized photoluminescence (PL) of the aforementioned bare films on quartz after photoexcitation at 470 nm. (e) Optical bandgap obtained from fits to the PL and electronic bandgap obtained from Elliot fits to the absorption spectra shown in Figure S10 for the aforementioned bare films on quartz. The error bars in parts b and e represent the 1 standard deviation range (68% confidence interval) for the respective fit parameters.

We are now in a position to start answering the question: Does any of the chlorine from the PbCl_2_ substitute iodide in a FA_1–*y*_Cs_*y*_Pb(I_1–*x*_Cl_*x*_)_3_ perovskite structure? Figure 2e shows a significant blue-shift in PL (Figure 2d) and bandgap energy of the FA_1–*y*_Cs_*y*_Pb(I_1–*x*_Cl_*x*_)_3_ films with increasing PbCl_2_ substitution, which is indicative of a slight change in perovskite composition. This widening of the bandgap could originate either from an increase in the Cs content at the perovskite A site or a Cl substitution for I at the X site. There have been many studies of Cs substitution of FA at the A site.^5,7^ However, since chloride ions have a significantly smaller ionic radius than iodide ions, it is not immediately clear if the X site substitution is possible. Indeed, there has been much debate previously as to what extent Cl can substitute I in the similar MAPb(I_1–*x*_Cl_*x*_) structure,^2,51−56^ with the most generous reports finding Cl can substitute only up to 3–4%.^51^ Notably, MAPb(I_1–*x*_Cl_*x*_)_3_ can be processed with a significant excess of chloride salts, yet most of the Cl is volatilized from the film in the form of MACl.^57,58^

Furthermore, we note that the formation of CsPbCl_3_ significantly complicates this system, as the latter will remove Cs from the bulk perovskite, causing a red-shift opposing a blue-shift from loss of FA or Cl substitution. Nevertheless, we can estimate the amount of substitution occurring for reasonable CsPbCl_3_ formation scenarios by estimating the content of either Cs or Cl incorporation that would be required to result in the observed bandgap shifts. Section 6 of the Supporting Information explores the bandgap trends that could be observed for varying levels of CsPbCl_3_ formation, Cl substitution, and Cl loss during film formation, assuming Vagard’s law to hold for I–Cl substitution from FA_1–*y*_Cs_*y*_PbI_3_ to FA_1–*y*_Cs_*y*_PbCl_3_. We find good agreement with our experimental trends for medium amounts of CsPbCl_3_ formation, when 40–80% of the evaporated chloride goes into CsPbCl_3_. Specifically, we find a blueshift of the bandgap owing to an FA/Cs compositional change is only possible for low amounts of CsPbCl_3_ formation and unreasonable amounts of FA loss. For example, the lower bound for FA loss required can be estimated by assuming that the blueshift is solely originating from Cs substitution with negligible CsPbCl_3_ formation. For the 30% PbCl_2_ substituted films, this would correspond to an increase in the Cs alloy fraction *y* by 0.12, requiring the loss of over half of the FA in the film. On the other hand, for the Cl substitution, the bandgap shift could be achieved with a Cl fraction *x* = 0.05, assuming half of the precursor Cl ends up in CsPbCl_3_.

To investigate whether Cl is actually present in our FA_1–*y*_Cs_*y*_Pb(I_1–*x*_Cl_*x*_)_3_ films, we performed X-ray photoemission spectroscopy (XPS) and energy dispersive X-ray spectroscopy (EDX) (Figures S15 and S16) measurements on the series of films. The XPS results displayed in Figure 2c are consistent with the presence of Cl in all the films apart from the Cl-free reference, and more Cl was detected in the films grown with a higher dose of PbCl_2_. Using EDX, we detected 11.5% Cl in a bare film of the champion 20% PbCl_2_ substitution, in very good agreement with the 13.3% Cl sublimed. This indicates that FACl is not significantly volatilized during processing (in contrast to MACl), matching the results from our modeling outlined above and described in detail within section 6 of the Supporting Information. While the Cl signal detected by XPS could have originated from either FA_1–*y*_Cs_*y*_Pb(I_1–*x*_Cl_*x*_)_3_, CsPbCl_3_ inclusions, or some amorphous phases, the absence of a CsPbCl_3_ XRD peak in the 10% PbCl_2_-substituted film (Figure 2a) combined with the presence of Cl 2*p* peaks in the XPS data (Figure 1c) are suggestive of incorporation of Cl in the FA_1–*y*_Cs_*y*_Pb(I_1–*x*_Cl_*x*_)_3_ perovskite phase. Furthermore, refinement of the lattice constant of the FA_1–*y*_Cs_*y*_Pb(I_1–*x*_Cl_*x*_)_3_ bulk perovskite phase shows a significant unit cell volume reduction with increasing PbCl_2_ substitution (Figure 2b, lattice refinement Figure S3), which is consistent with the formation of a FA_1–*y*_Cs_*y*_Pb(I_1–*x*_Cl_*x*_)_3_ phase with a small but increasing Cl alloy fraction *x*. We note the estimate for *x* that we calculated above is only 5%, and since we observe significant Cl present, this suggests that there is also significant CsPbCl_3_, in agreement with our simple modeling outlined above or other Cl-containing amorphous phases. The increase in the lattice constant and drop in bandgap for the 40% PbCl_2_ sample can be explained by the gradually increasing amount of CsPbCl_3_ eventually removing Cs and Cl from the bulk perovskite, as confirmed by modeling of the kinetics of FA_1–*y*_Cs_*y*_Pb(I_1–*x*_Cl_*x*_)_3_ formation as a function of PbCl_2_ substitution (detailed in section 6 of the Supporting Information). A detailed description of the lattice refinement method and Elliot fitting method can be found in sections 2.1 and 5.2 of the Supporting Information, along with additional characterization such as film stability (Figures S4 and S5) and top-down and cross-sectional scanning electron microscopy (SEM) images (Figures S7 and S8).

Given the significance of nonradiative losses to solar-cell performance, we now examine how the dose of PbCl_2_ during growth affects the PL lifetime of charge-carrier lifetimes and PLQY of this series of films. Stretched exponential fits to these time-resolved photoluminescence (TRPL) decays (Figure S12) reveal an increase of over an order of magnitude in PL lifetime even when only a small fraction of Cl is added (Figure 3a). We observe a similar trend for PLQY of neat films (Figure 3a), which show a jump from values close to our detection limit of 0.02% for the neat iodide films to 0.3% for the 40% PbCl_2_ processed film. Together, these measurements clearly demonstrate that processing with fractional substitution of PbI_2_ for PbCl_2_ has an effect of reducing nonradiative recombination in the coevaporated FA_0.83_Cs_0.17_PbI_3_ films. This may be due to fewer crystalline defects being present in the film grown with PbCl_2_ or an active passivation effect of the Cl ions. The order of magnitude increase in photocarrier lifetimes is consistent with that of vapor deposited MAPbI_3_^43,44^ and MAPbI_3–*x*_Cl_*x*_,^49,56^ though even the latter only displayed TRPL lifetimes up to 83 ns.^49^

**Figure 3:**
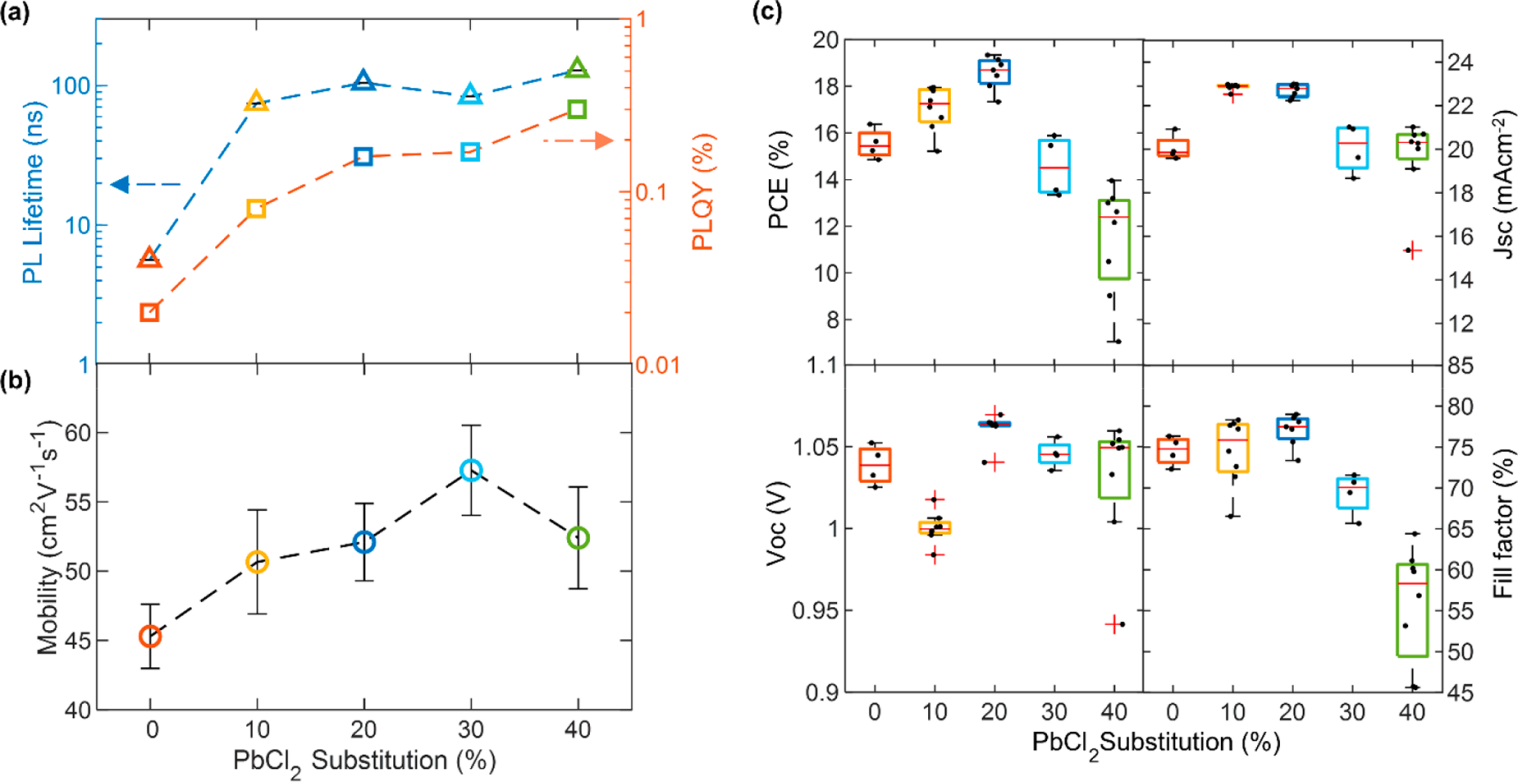
Evidence for defect passivation of FA_1–*y*_Cs_*y*_Pb(I_1–*x*_Cl_*x*_)_3_ thin films with Cl addition. (a) Photocarrier lifetimes (blue, left) and photoluminescence quantum yield (PLQY) (red, right) of FA_1–*y*_Cs_*y*_Pb(I_1–*x*_Cl_*x*_)_3_ thin films on z-cut quartz, grown with various amounts of PbI_2_ substituted with PbCl_2_. The lifetimes were obtained from stretched exponential fits to time-resolved photoluminescence (TRPL) traces, which were obtained by photoexciting the samples with a 1 MHz pulsed 470 nm laser with a fluence of 20 μJ/cm^2^. The PLQY was obtained through 532 nm photoexcitation at 24 mW cm^–2^ intensity. (b) Charge-carrier mobilities obtained from optical-pump THz-probe spectroscopy done on the aforementioned bare films. Additional experimental details can be found in the Supporting Information. (c) Current–voltage (*J*–*V*) characteristics for reverse scans of 0.25 cm^2^ and 1 cm^2^ devices made from the aforementioned FA_1–*y*_Cs_*y*_Pb(I_1–*x*_Cl_*x*_)_3_, tested under simulated AM1.5 solar irradiance. The solar cells were made with the following p-i-n structure ITO/PTAA/perovskite/C60/BCP/Ag.

Figure 3b shows that there is a monotonic increase in FA_1–*y*_Cs_*y*_Pb(I_1–*x*_Cl_*x*_)_3_ charge-carrier mobility when increasing the PbCl_2_ fraction up to 30%, with the charge-carrier mobility increasing from 46 cm^2^/(V s) for the neat iodide perovskite to 56 cm^2^/(V s) for the sample made with 30% PbCl_2_. These findings imply that the growth of the higher bandgap ∼3.0 eV CsPbCl_3_ phase^59,60^ does not introduce additional charge-carrier traps in the film. In fact, CsPbCl_3_ may even play a role in passivating these defects, specifically because it has a higher bandgap and seemingly forms a good interface with the iodide bulk perovskite.

We now investigate how different doses of PbCl_2_ and related improvement in crystallographic and optoelectronic properties of FA_1–*y*_Cs_*y*_Pb(I_1–*x*_Cl_*x*_)_3_ affects the performance of solar cells. Figure 3c shows boxplots for *J*–*V* scans obtained from solar cells made using the PbCl_2_-substituted perovskite compositions, demonstrating significant enhancement in all *J*–*V* characteristics for the midrange of PbCl_2_ substitution, as highlighted in Figure 1c (*J*–*V* curves Figure S20). While performance initially improves as more PbCl_2_ is added, the PCE of the devices drops after 20% PbCl_2_ substitution, primarily driven by a reduction in FF. In addition, we observed that, surprisingly, some of the 30% PbCl_2_ devices showed a phase instability under illumination, resulting in significantly reduced performance. These devices are not included in Figure 3c but are shown in Figure S22. Overall, the initial boost in both *J*_SC_ and *V*_OC_ with PbCl_2_ substitution are consistent with the reduced nonradiative recombination, increased absorption coefficient, increased charge-carrier lifetime, and improvement in charge-carrier mobility.

Surprisingly, there is a substantial drop in *V*_OC_ for the 10% PbCl_2_ sample, in spite of having evidence of reduced nonradiative recombination from higher TRPL lifetimes and PLQY. We associate this drop with the poor MHP phase formation for the 10% PbCl_2_ during deposition resulting in a worse bottom interface. Indeed, in contrast to all other compositions, the 10% PbCl_2_ is not phase-stable before annealing, implying significant recrystallization during the annealing step, which could significantly damage the bottom interface and hence reduce *V*_OC_. The reduced nonradiative recombination in the bulk material still leads to the expected improvement in *J*_SC_ by allowing more charge carriers to reach the interfaces.

It is worth noting that even though optoelectronic properties generally continue to improve for higher Cl concentrations, this is not reflected in the PCE, largely owing to a significant drop in FF. The main culprit for this drop is likely to be the increased presence of CsPbCl_3_ in the higher percentage PbCl_2_ films. Indeed while optoelectronic properties show CsPbCl_3_ to be benign or beneficial for reducing nonradiative recombination, inclusions of higher bandgap CsPbCl_3_ will act as high-energy obstacles for charge-carrier transport in the surrounding iodide-rich perovskite phase, hence limiting device performance. As such, we conclude that the optimum amount of PbCl_2_ substitution is around 20%, when small inclusions of a CsPbCl_3_ phase just begin to form. This is in a good agreement with a series of films where the PbCl_2_ was introduced as a thin interlayer before the FA_0.83_Cs_0.17_PbI_3_ perovskite, which also show improved PCE when CsPbCl_3_ first starts to form (Figures S18 and S19), and the recent results of Min et al., showing the benefits of a thin FASnCl_*x*_ layer at the ETL.^61^

In conclusion, we demonstrate PbCl_2_ as a viable *in situ* defect passivant for FA_0.83_Cs_0.17_PbI_3_ vapor deposited MHP solar cells, effective both as an interlayer before deposition and deposited in the bulk. We observed Cl retention with the as-processed films and show evidence of a small fractional substitution of Cl at the X site of the perovskite crystal. The maximum uptake is observed for 30% PbCl_2_ processed film exhibiting a Cl substitution in the perovskite crystal of ∼5%, resulting in an electronic bandgap of ∼1.6 eV. In general, PbCl_2_ substitution results in films of excellent optoelectronic properties. These films show extremely low energetic disorder and exhibited improved crystallinity as well as increased radiative efficiency, PL lifetimes, and charge-carrier mobility. These enhancements led to solar cells with significantly improved PCE of up to 19.3% (18.7% steady-state) for devices with 20% PbCl_2_ substitution. We expect that our work will lay the foundations for incorporating Cl into vapor deposited perovskite PV cells and expect that further efficiency improvements may result from extending this approach to Br-based MHP to yield highly efficient, passivated, vacuum deposited multijunction solar cells.

To date the performance of MHP solar cells fabricated via vapor-deposition has lagged behind solution-processed cells, despite vapor deposition being a process that can easily deliver highly conformal MHP thin films. This has largely been a consequence of the much shorter charge-carrier lifetimes typically observed in vapor-deposited MHP films. Our work here has shown a clear and effective strategy to achieving the highest quality thin films from vapor deposition and will lead to significant improvements in the performance of vapor-deposited MHP solar cells in the near future. Moreover, this “dry” solvent-free processing methodology is directly compatible with high-volume manufacturing.

## Supporting Information Available

Additional details about device fabrication and experimental procedures as well as SEM images, XRD stability measurements, lattice refinement details, OPTP spectra and fits, a detailed investigation of the amount of Cl incorporation, as well as additional experiments, such as the use of a thin PbCl_2_ interlayer (PDF)
