# Supplementary material for: Solvent-Free Method for Defect Reduction and Improved Performance of p-i-n Vapor-Deposited Perovskite Solar Cells
Source: ACS Energy Lett. 2022 May 9;7(6):1903–11. doi: 10.1021/acsenergylett.2c00865 (PMC9199003; doi:10.1021/acsenergylett.2c00865)
Supplement: Supplementary file 1 — nz2c00865_si_001.pdf [file nz2c00865_si_001.pdf]

# Supplementary Information for:
# Solvent-Free Method for Defect Reduction and Improved Performance of p-i-n Vapor-Deposited Perovskite Solar Cells

*Kilian B. Lohmann[1], Silvia G. Motti[1], Robert D. J. Oliver[1], Alexandra J. Ramadan[1], Harry C. Sansom[1], Qimu Yuan[1], Karim A. Elmestekawy[1], Jay B. Patel[1], James M. Ball,[1] Laura M. Herz[1,2], Henry J. Snaith[1], and Michael B. Johnston*[1]*

*[1]Department of Physics, University of Oxford, Clarendon Laboratory, Parks Road, OX1 3PU, United Kingdom*

*[2]Institute for Advanced Study, Technical University of Munich, Lichtenbergstrasse 2a, D-85748 Garching, Germany*

E-mail: michael.johnston@physics.ox.ac.uk

# Table of Contents

1. Device Fabrication ........ 3
1.1 Bottom Contact ........ 3
1.2 P-Type Contact Layer ........ 3
1.3 Perovskite Absorber Layer ........ 3
1.4 N-Type Contact Layer ........ 3
1.5 Top Contact ........ 4
1.6 Current-Voltage Characterization ........ 4
2. X-ray Diffraction Measurements and Analysis ........ 4
2.1 Lattice Refinement ........ 6
2.2 XRD Stability Measurements ........ 8
3. X-ray Photoemission Spectroscopy ........ 9
4. Scanning Electron Microscopy ........ 10
4.1 Energy Dispersive X-ray Spectroscopy ........ 12
5. Spectroscopic Measurements ........ 13
5.1 Absorption Coefficient and Bandgap ........ 13
5.2 Elliott Fits ........ 14
5.3 External Quantum Efficiency and Urbach Energy ........ 16
5.4 Photoluminescence Spectra ........ 17
5.5 Time-Resolved Photoluminescence ........ 17
5.6 Optical Pump THz Probe Spectroscopy ........ 18
5.7 Photoluminescence Quantum Yield ........ 21
6. Cl Incorporation ........ 21
6.1 Theory ........ 21
6.2 Evidence for Cl in the Final Films ........ 25
6.3 Evidence for FACl Loss ........ 26
6.4 Importance of $CsPbCl_3$ Formation ........ 28
7. Extra J-V Data ........ 30
8. Effect of annealing ........ 33
References ........ 34

# 1. Device Fabrication

## 1.1 Bottom Contact

Films were deposited on indium-doped tin oxide (ITO) glass (AMG). The substrates were cleaned using industrial detergent Decon90 (1% vol in de-ionised water), de-ionised water, acetone, and isopropyl alcohol. Immediately prior to deposition, the substrates were cleaned under UV-ozone for 15 minutes.

## 1.2 P-Type Contact Layer

Poly[bis(4-phenyl)(2,4,6-trimethylphenyl)amine (PTAA) (Xi'an Polymer Light Technology) was spin-coated in an $N_2$ glovebox. PTAA was dissolved in toluene at a concentration of 1.5 mg/mL; 100 µL of solution was applied statically onto the substrate, which was then spun up to 6000 rpm for 30 s, with an acceleration of 2000 rpm/s. After deposition, the substrate with PTAA was annealed in a $N_2$ glovebox at 100 °C for 10 mins.

## 1.3 Perovskite Absorber Layer

$CH(NH_2)_2$ (FAI) (Greatcell), $PbI_2$ (99.998% metal base, Alfa-Aeser), CsI (99.998% metal base, Alfa-Aeser), and $PbCl_2$ (99.998% metal base, Alfa-Aeser) were co-evaporated in a custom-built thermal evaporator chamber to deposit compositions of the form $FA_{1-y}Cs_yPb(I_{1-x}Cl_x)_3$ by progressively substituting $PbI_2$ with $PbCl_2$. Specifically, starting with the neat-iodide aimed composition of $FA_{0.83}Cs_{0.17}PbI_3$, we substituted 0%, 10%, 20%, 30%, 40% of the molar deposition rate of $PbI_2$ with $PbCl_2$, respectively. The FAI and CsI rates were kept the same for all deposition. During the evaporation, the pressure was typically < $1 \times 10^{-6}$ mbar. The source rates were kept constant using gold-plated quartz microbalances (QCM) and PID-loop-control software, with set rates and typical deposition temperatures detailed in Table S1. The sublimation rate of the inorganic precursors was controlled using QCMs adjacent to the crucible, while the FAI sublimation rate was controlled using a QCM close to the substrate, following the method we previously used for MAI [1]. Prior to every deposition, the FAI crucible was topped up to 1.1 g, adding fresh FAI to the remainder of previous deposition. For a typical 500 nm perovskite deposition, we evaporated 0.21 – 0.23 g of FAI. Our chamber is set up such that the walls of the chamber are cooled to 17°C, while the substrate was held constant at 20°C.

Unless specified otherwise, all samples were annealed after deposition in an $N_2$ glovebox for 30 minutes at 135 °C.

| Material | FAI | CsI | $PbI_2$ | $PbCl_2$ | $C_{60}$ | BCP |
|---|---|---|---|---|---|---|
| Set rate (Å/s) | 0.2 | 0.04 | 0.29-0.17 | 0-0.072 | 0.06 | 0.1 |
| Temperature (°C) | 150-165 | 390-410 | 265-280 | 305-340 | 350-390 | 140-160 |

**Table S1:** Set rate values for the materials vapor deposited for this work, as well as the range of temperatures typically reached by the crucibles during deposition

## 1.4 N-Type Contact Layer

$C_{60}$ fullerene (99.9%, Acros Organics) was vacuum deposited in the same custom thermal evaporator chamber to 25 nm at 0.06 Å/s. The rate was controlled using a gold-plated quartz microbalance. Following $C_{60}$ deposition, without breaking vacuum, 2 nm of Bathocuproine (BCP) (99.5%, Sigma-Aldricht) were deposited in the same evaporator chamber at 0.1 Å/s.

### 1.5 Top Contact

100 nm of Ag was thermally evaporated in a commercial Lesker nano36 chamber, starting at a rate of 0.2 Å/s before ramping to 1.5 Å/s, controlled by a gold-plated quartz microbalance. A mask was used such that the entire photoactive area of the devices was covered.

### 1.6 Current-Voltage Characterization

Devices were tested in ambient atmosphere using a Wavelabs Sinus-220 solar simulator and a Keithly 2400 sourcemeter at AM1.5 100 mW/cm$^2$ sunlight. The intensity of the solar simulator was set to produce the same short-circuit current from a KG3-filtered silicon reference photodiode (Fraunhofer) as its certified value. The active area illuminated for each device was 0.25 or 1 cm$^2$, which was defined by black anodized metal masks. The measurement routine for each device was as follows: first, the open-circuit voltage ($V_{OC}$) was measured for 3 s under illumination; then the current-voltage characteristics under illumination were measured scanning first in the reverse scan direction, from 1.2 – 0 V, followed immediately by the forward scan, at a scan rate of 0.013 V/s in each direction; next the steady-state maximum power output under illumination was estimated using a maximum point tracking algorithm (gradient descent) over 30 s; finally the short-circuit current ($J_{SC}$) was recorded over a period of 3 s. The devices tended to improve slightly over the course of 1 week after deposition. All J-V data shown in this work was measured 1 week after deposition of the top contact. We tested 8 devices for each composition other than the control 0% $PbCl_2$ devices, which had 4 devices in this particular batch. The J-V characteristics shown in Figure 3c of the main text do not show shorted devices, defined as having $J_{SC}$ < 1 mA/cm$^2$; there was one such device from all the measurements we took.

## 2. X-ray Diffraction Measurements and Analysis

X-ray diffraction measurements (XRD) were performed on a Panalytical X'pert powder diffractometer equipped with a copper X-ray source (Cu-Kα 1.54 Å source set to 40 kV and 40 mA). The data were corrected for sample displacement by shifting the *x*-axis of the individual profiles such that the reference ITO peak sits at 2θ = 30.4°.

To obtain the peak position, and peak intensity of the $PbI_2$ (001) peak at 12.7°, and the (100) $CsPbCl_3$ peak at 15.8°, we fit a pseudo-Voigt distribution to the data. The pseudo-Voigt distribution was defined as described by Thompson et al. [2].

Figure 2a in the main text only shows a small section of the XRD trace. Figure S1 shows the full spectra for each of the samples, normalized with respect to their highest peak. As discussed in the main text, all films are made to have a small excess of lead-halide. We find this results in a small excess $PbI_2$ peak for all amounts of Cl substitution, as shown in Figure S2. We believe the $PbI_2$ excess drops initially because of the overall reduction in $PbI_2$ sublimated, while the subsequent increase is due to the formation and re-evaporation of FACl at the substrate, as discussed in Section 6.3. Figure S2 also shows how the XRD peak intensity of the $CsPbCl_3$ peak increases linearly with added Cl, for 20% $PbCl_2$ substitution onwards.

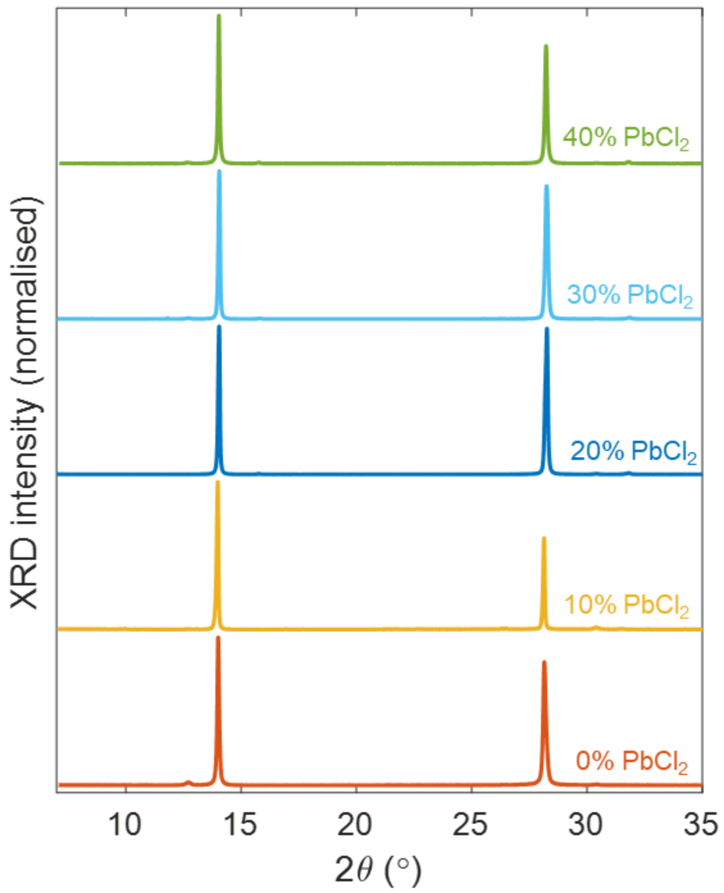


**Figure S1:** X-ray diffraction patterns of vapor deposited $FA_{1\text{-}y}Cs_yPb(I_{1\text{-}x}Cl_x)_3$ full devices after testing, grown with varying amounts of the $PbI_2$ substituted for $PbCl_2$. Each trace has been normalized with respect to its highest peak.

**(a)**

| $PbCl_2$ substitution | XRD intensity /arb. units $PbI_2$ | $CsPbCl_3$ |
|---|---|---|
| 0% | 3.85 | 0.00 |
| 10% | 0.23 | 0.00 |
| 20% | 0.12 | 1.02 |
| 30% | 3.00 | 2.13 |
| 40% | 2.01 | 2.54 |

**(b)**

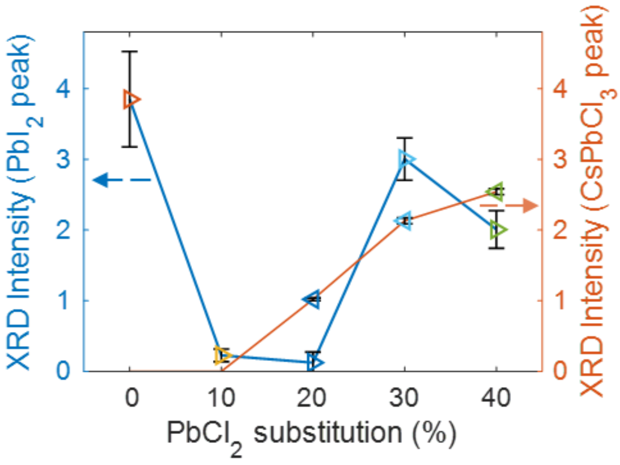


**Figure S2: (a)** Table showing the intensity of the $PbI_2$ (001) peak and $CsPbCl_3$ (100) peak of vapor deposited $FA_{1\text{-}y}Cs_yPb(I_{1\text{-}x}Cl_x)_3$ full devices after testing, grown with various amounts of $PbI_2$ substituted for $PbCl_2$ $PbI_2$ and $CsPbCl_3$ intensity. These values were obtained from pseudo-Voigt fits to the corresponding XRD peaks and normalized with respect to the ITO peak of each sample. These values graphically represented in **(b)**

### 2.1 Lattice Refinement

Firstly, the XRD pattern for the 0% sample was fitted with a cubic perovskite phase (space group $Pm\bar{3}m$), $PbI_2$ (space group $P\bar{3}m1$) [3] and ITO (space group $Ia\bar{3}$) [4] using a Pawley fit implemented via TOPAS-Academic V6 (Figure S3). The cubic perovskite phase refined to a lattice parameter of a = 6.350(2) Å, which is smaller than α-(FA)$PbI_3$ (a = 6.3620(8) Å) [5] and larger than α-$CsPbI_3$ (a = 6.2894(2) Å at 634 K ) [6], as expected. The lattice parameters of $PbI_2$ refined to a = 4.542(5) Å and c = 6.997(2) Å compared to a = 4.54 Å and c = 6.94 Å reported on the ICSD [3]. The ITO phase refined to a lattice parameter of a = 10.202(3) Å, which is larger than the lattice parameter reported for 3-6% Sn-doped $In_2O_3$ on the ICSD (10.1234(2) Å to 10.1328(1) Å) [4]. We do not know the exact composition of our ITO glass coating. The ITO lattice parameter a = 10.202(3) Å was fixed in the subsequent fittings for the other samples in the series to use as an internal reference for comparing lattice parameters of the cubic perovskite phase. For each XRD pattern a zero error (linear θ offset), specimen displacement error (cos(θ)-dependent offset) and tilting error (peak shape modifier) were refined.

In all cases, the perovskite phase was the majority phase but careful inspection, when plotted on a logscale, revealed the presence of peaks due to impurity phases (Figure S3). Over the series the δ-$FAPbI_3$, δ-$CsPbI_3$, $CsPbCl_3$, and $PbI_2$ impurity phases were identified. In Figure S3, coloured tick marks have been used to identify the phases used in the fittings. The $PbI_2$ is ever-present as expected from the excess $PbI_2$ deposited (red tick marks). δ-$CsPbI_3$ is present in the 0% and 10% $PbCl_2$ samples (green tick marks). A sharp peak due to δ-(FA)$PbI_3$ is observed in the 30% $PbCl_2$ sample (marked with +). The $CsPbCl_3$ phase is present in samples above 20% $PbCl_2$ substitution (blue tick marks).

| **$PbCl_2$ Substitution** | **Lattice Parameter /Å** | **Error /Å** |
|---|---|---|
| 0% | 6.35021 | 0.00173 |
| 10% | 6.34633 | 0.00142 |
| 20% | 6.32808 | 0.000912 |
| 30% | 6.32710 | 0.00117 |
| 40% | 6.33185 | 0.000657 |

**Table S2:** Cubic lattice parameter for the bulk perovskite phase of vapor deposited $FA_{1-y}Cs_yPb(I_{1-x}Cl_x)_3$ full devices grown with varying amounts of $PbI_2$ substituted for $PbCl_2$. The values were obtained through a Pawley fit (Figure S3) to the XRD patterns shown in Figure 2a and S1.

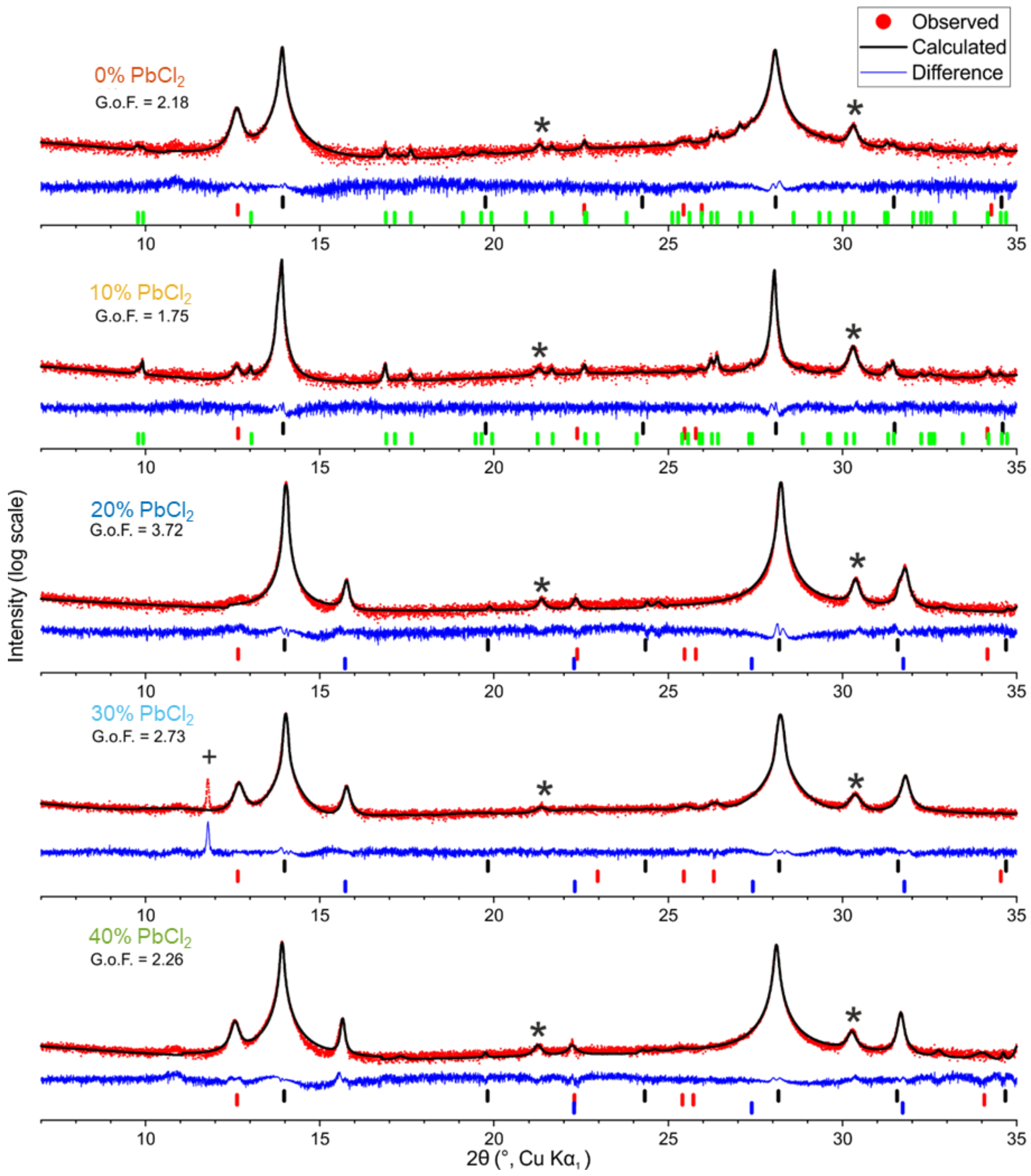


**Figure S3:** The XRD data (red circles) fitted with a Pawley fit (black line) and the difference (blue line) plotted on a log scale to show impurity phases. Tick marks below the difference line show the peak positions of phases included in the fits; cubic perovskite (black), $PbI_2$ (red), δ-$CsPbI_3$ (green), $CsPbCl_3$ (blue). The peaks due to ITO are marked with * and were included in the fit. A peak due to a δ-(FA)$PbI_3$ impurity which could not be included in the fit is marked with +.  All peaks are accounted for.

### 2.2 XRD Stability Measurements

We investigated the phase stability of the perovskite compositions outlined in this study by measuring the XRD of bare films grown on ITO/PTAA over the course of 30 days, with the films stored in ambient air in the dark (40-50% relative humidity). Figure S4 and S5 show this data for films before and after annealing, respectively. Note that the unannealed 10% $PbCl_2$ substitution film proved particularly unstable, and turned completely yellow after 1 week, so this data point is shown instead.

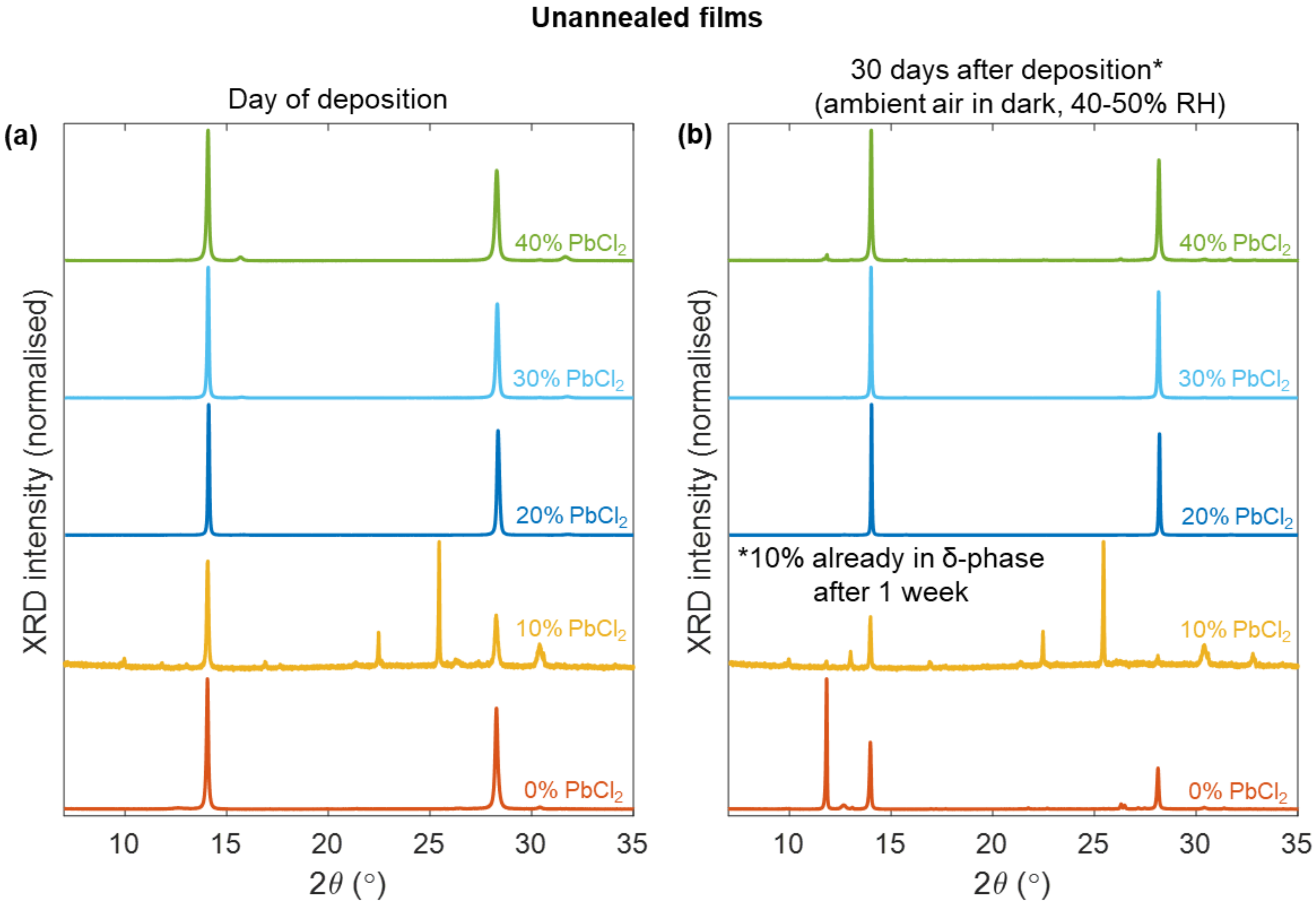


**Figure S4:** X-ray diffraction patterns of vapor deposited $FA_{1-y}Cs_yPb(I_{1-x}Cl_x)_3$ thin films grown with varying amounts of $PbI_2$ substituted for $PbCl_2$, on ITO/PTAA. The films were not annealed before or after measurement. **(a)** shows the films on the day of deposition, **(b)** shows the films after 30 days stored in the dark, in ambient air (40-50% RH), apart from the 10% $PbCl_2$ film, which is shown already after 1 week.

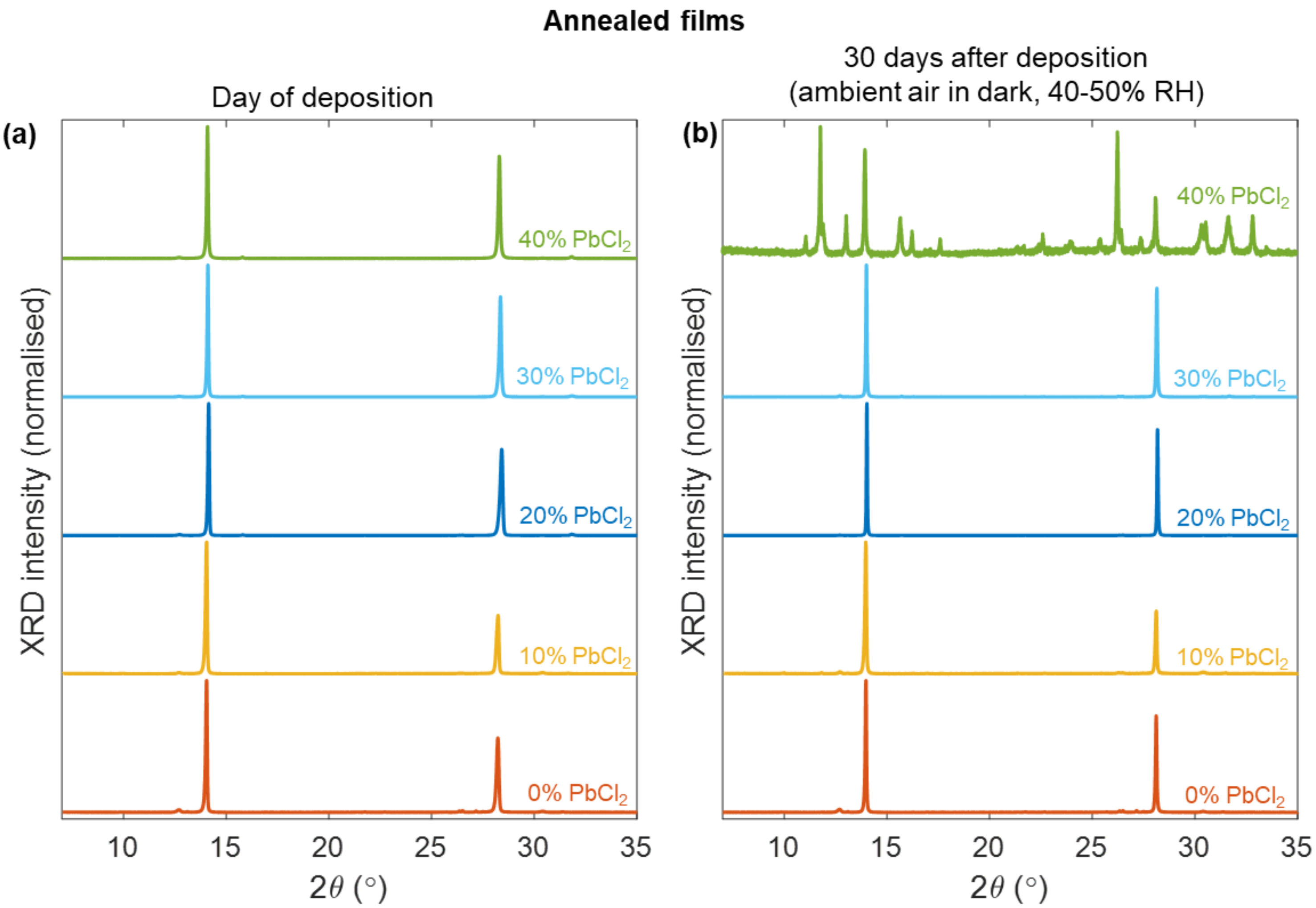


**Figure S5:** X-ray diffraction patterns of vapor deposited $FA_{1-y}Cs_yPb(I_{1-x}Cl_x)_3$ thin films grown with varying amounts of $PbI_2$ substituted for $PbCl_2$, on ITO/PTAA. After deposition, the films were annealed for 30 mins at 135°C. **(a)** shows the films on the day of deposition, **(b)** shows the films after 30 days stored in the dark, in ambient air (40-50% RH)

## 3. X-ray Photoemission Spectroscopy

X-ray photoemission spectroscopy measurements were carried out using a Thermo Scientific Kα X-Ray Photoelectron Spectrometer with a monochromatic Al Kα X-Ray source at a take-off angle of 90 degrees. The analysis area was 200 µm x 200 µm and the core level spectra were recorded using a pass energy of 20 eV (an approximate resolution of 0.4 eV). Fitting procedures to extract peak positions from the XPS data were carried out using Casa XPS software suite. A Shirley background was used, and the spectra were fit with a mixture of Gaussian/Lorentzian (Lorentzian = 30%) line shapes.

Figure 2c of the main text shows the high-resolution scan of Cl 2*p* region. Figure S6a shows the XPS survey scans, and Figure S6b shows the corresponding calculated relative atomic composition. The numerical values corresponding to Figure S6b are shown in Table S3.

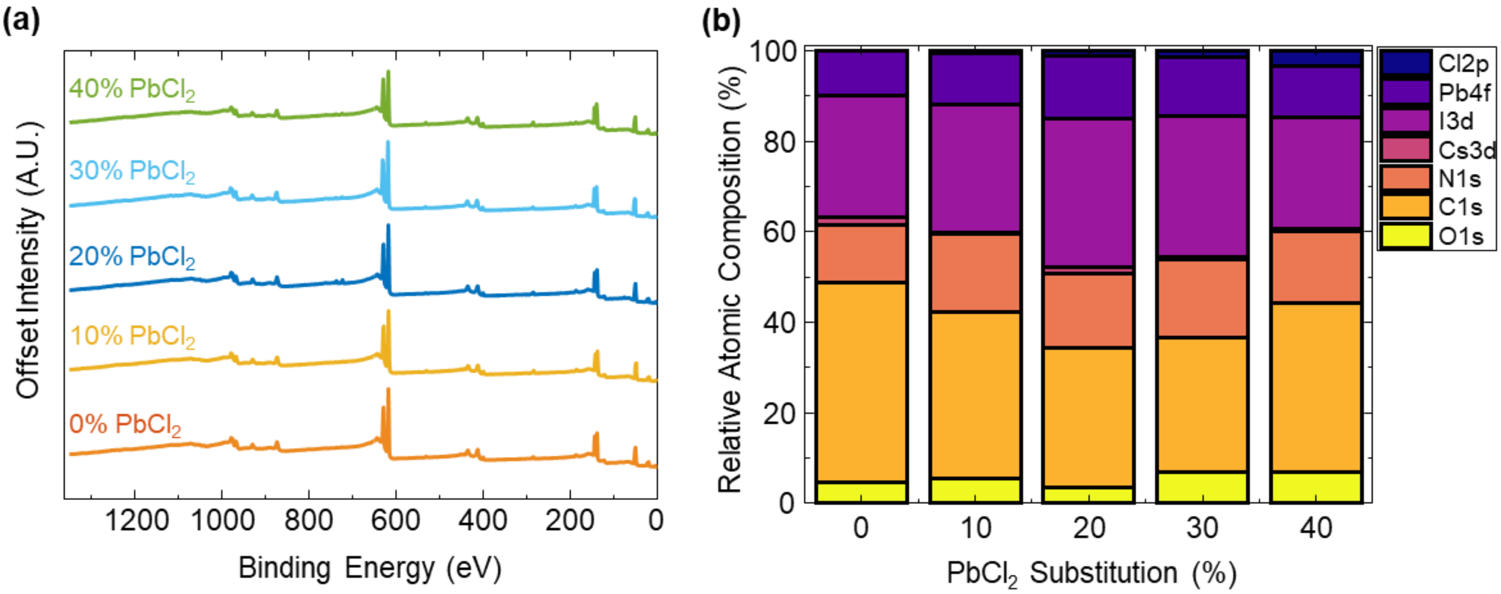


**Figure S6: (a)** X-ray photoemission spectroscopy (XPS) spectra of vapor-deposited $FA_{1-y}Cs_yPb(I_{1-x}Cl_x)_3$ thin films grown with varying amounts of $PbI_2$ substituted for $PbCl_2$, as denoted on top of each curve, on ITO/PTAA. **(b)** Relative atomic composition calculated from the XPS scans

| $PbCl_2$ Substitution | Relative Atomic Composition /% O1s | C1s | N1s | Cs3d | I3d | Pb4f | Cl2p |
|---|---|---|---|---|---|---|---|
| 0% | 4.5(4) | 44.1(8) | 12.7(5) | 1.74(6) | 26.8(3) | 10.1(2) | 0 |
| 10% | 5.5(4) | 36.8(9) | 17.5(5) | 0.09(0.01) | 28.1(4) | 11.4(3) | 0.6(2) |
| 20% | 3.4(5) | 31(1.2) | 16.4(6) | 1.44(7) | 32.8(5) | 13.7(4) | 1.2(4) |
| 30% | 6.9(5) | 30(1.1) | 17.3(6) | 0.47(5) | 31.2(4) | 13.1(4) | 1.4(5) |
| 40% | 7.0(4) | 37(1) | 16.0(5) | 0.48(4) | 24.7(3) | 11.2(3) | 3.4(5) |

**Table S3:** Numerical values for the relative atomic composition percentage illustrated in Figure S6b. The value in parentheses represents the standard deviation from the fit to the XPS spectra used to obtain the relative atomic composition.

## 4. Scanning Electron Microscopy

Figure S7 and S8 show typical top down and cross-section scanning electron microscopy (SEM) images for the series of films investigated in this study. The SEM images were taken on an FEI Quanta 600 FEG, at 5 and 10 keV acceleration voltage for top-down and cross-section images respectively, with current defined by spot size 2.5. Prior to measurement, the SEM chamber was pumped down to high vacuum (< $2x10^{-4}$ mbar).

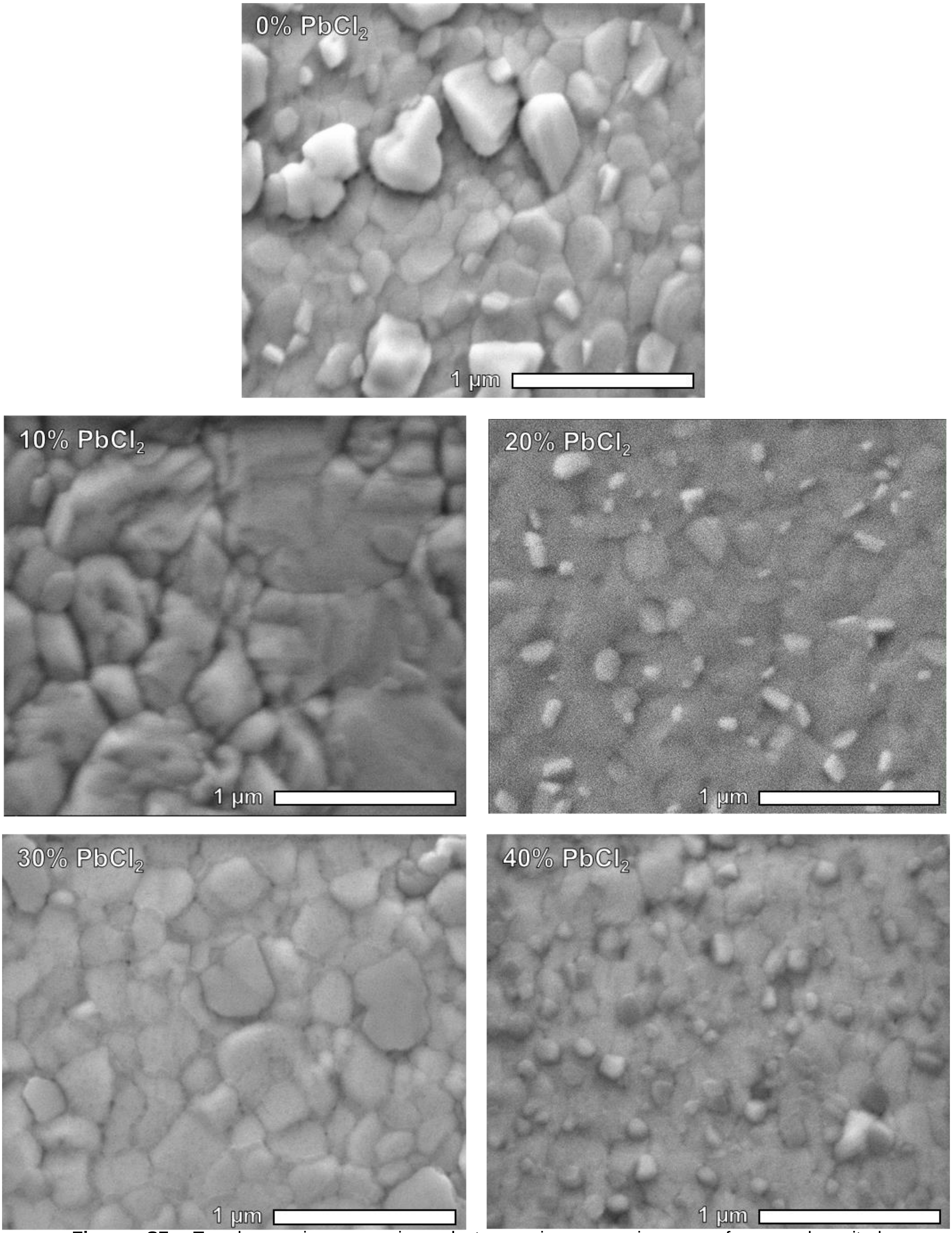


**Figure S7:** Top-down view scanning electron microscope images of vapor-deposited $FA_{1-y}Cs_yPb(I_{1-x}Cl_x)_3$ thin films on ITO/PTAA, grown with varying amounts of $PbI_2$ substituted for $PbCl_2$, as denoted in the top left corner of each image. The scale bars at the bottom left of each image are 1 µm long.

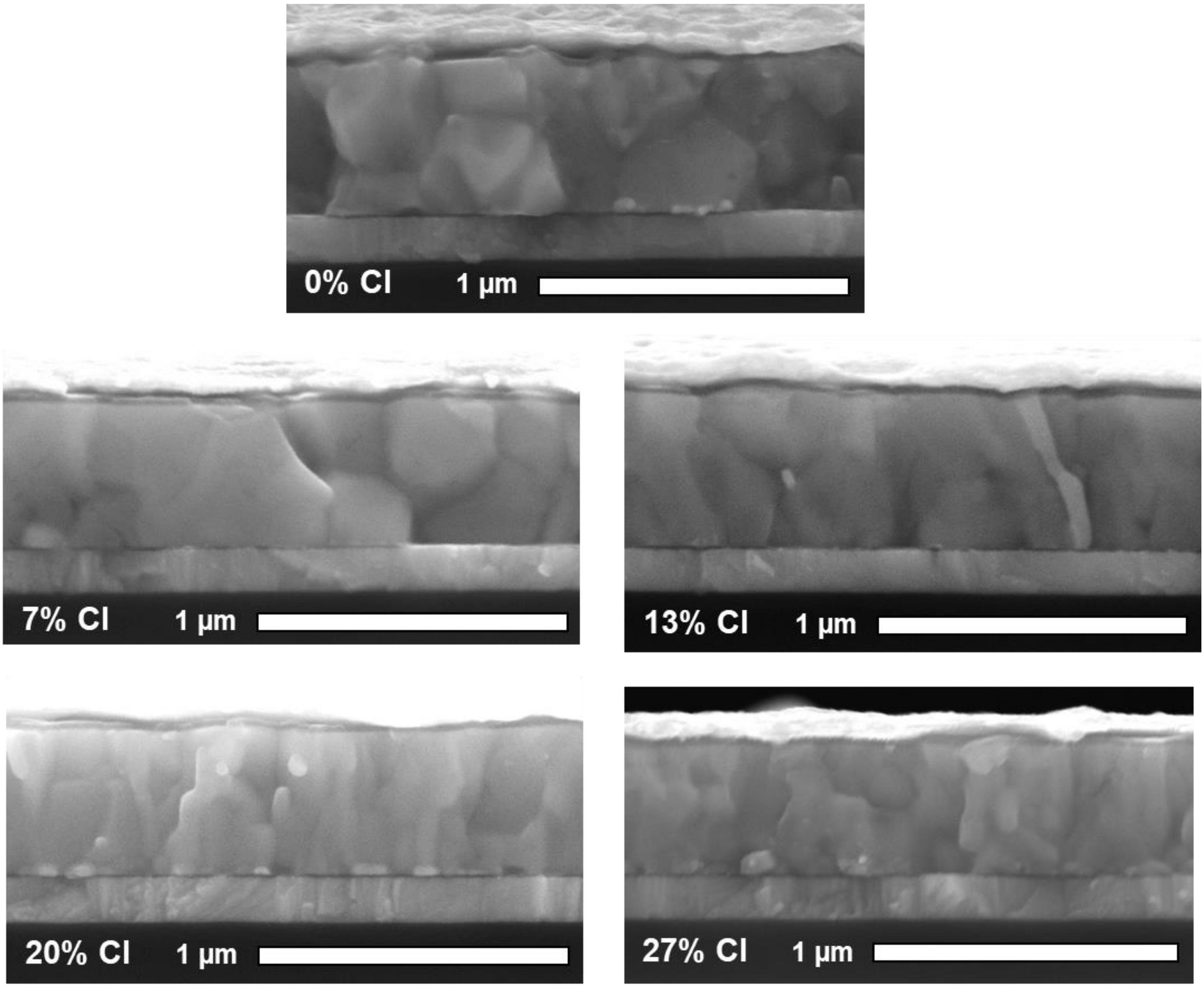


**Figure S8:** Cross-section view scanning electron microscope images of vapor-deposited $FA_{1-y}Cs_yPb(I_{1-x}Cl_x)_3$ full devices, grown with varying amounts of $PbI_2$ substituted for $PbCl_2$, as denoted in the bottom left corner of each image. The scale bars at the bottom left of each image are 1 µm long.

### 4.1 Energy Dispersive X-ray Spectroscopy

We performed SEM energy dispersive x-ray spectroscopy (EDX) on the same system described above, using an Oxford Instruments detector. For each sample, we took 9 scans over areas of order 0.01 $mm^2$, over a 90 s duration, using a 10 keV electron beam. To correct for differential Cl and I detection, we also prepared 500 nm thick samples of just $PbI_2$ and $PbCl_2$. We measured the EDX spectra for samples from the set of devices presented in the main text (thin films capped with $C_{60}$/BCP), as well as two thin films grown with 20% $PbCl_2$, before and after annealing. Table S4 shows the average relative atomic composition detected for the reference $PbI_2$ and $PbCl_2$ samples, and the calculated correction factor for I and Cl. Table S5 shows the corrected relative atomic compositions for the 20% $PbCl_2$ thin films and the series of devices, normalized to the Pb detected.

| Reference sample | Relative Atomic Composition /% Pb | I | Cl | Correction factor |
|---|---|---|---|---|
| $PbI_2$ | 33.649 | 66.351 | n/a | 1.0141 |
| $PbCl_2$ | 32.232 | n/a | 67.768 | 0.9507 |

**Table S4:** Relative atomic composition measured using energy-dispersive X-ray spectroscopy (EDX) for the 500 nm $PbI_2$ and $PbCl_2$ reference sample, and the corresponding correction factor

| $PbCl_2$ Substitution | Corrected Relative Atomic Composition (normalized) Pb | I | Cl | Measured Cl/(I+Cl) /% |
|---|---|---|---|---|
| 20% as deposited | 1.00(2) | 2.64(4) | 0.43(4) | 14.0(1.2) |
| 20% annealed | 1.00(2) | 2.69(4) | 0.35(3) | 11.5(1.2) |
| 0% device | 1.00(2) | 2.96(2) | n/a | 0 |
| 10% device | 1.00(4) | 3.09(4) | 0.12(4) | 3.8(1.2) |
| 20% device | 1.00(4) | 2.96(3) | 0.20(5) | 6.2(1.5) |
| 30% device | 1.00(3) | 2.97(5) | 0.27(3) | 8.2(0.9) |
| 40% device | 1.00(2) | 2.63(5) | 0.66(4) | 20.0(1.3) |

**Table S5:** Relative atomic composition measured using energy-dispersive X-ray spectroscopy (EDX) for 500 nm films of $FA_{1-y}Cs_yPb(I_{1-x}Cl_x)_3$ grown with varying amounts of $PbI_2$ substituted for $PbCl_2$. The atomic composition values have been corrected using the factors derived in Table S4, and normalized to the measured Pb content. The table entries labeled as device were all annealed, and capped by $C_{60}$/BCP.

## 5. Spectroscopic Measurements

### 5.1 Absorption Coefficient and Bandgap

Transmission and reflection measurements were taken using a modified Bruker Vertex 80v Fourier Transform Interferometer (FTIR), using a near-infrared source, $CaF_2$ beam splitter, and Si diode detector. The absorption coefficient was obtained using the formula $\alpha = log_{10}\left(\frac{1-R}{T}\right)/t$, where $R$ is the amount of light reflected off the sample, $T$ is the amount of light transmitted through the sample, and $t$ is the film thickness. Figure S9 shows an example of the transmission and reflection data for the 20% $PbCl_2$ sample, showing the oscillations in R and T indicative of a smooth film. Figure S10 shows the absorption coefficient for all $PbCl_2$ compositions investigated in this work. The electronic bandgap was extracted from the absorption edge using Elliott fits, described in the next section.

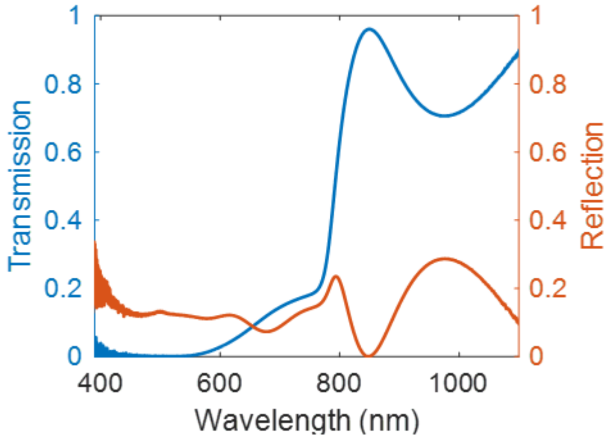


**Figure S9:** Transmission (blue) and reflection (red) spectra of vapor-deposited $FA_{1-y}Cs_yPb(I_{1-x}Cl_x)_3$ thin films grown with 20% of $PbI_2$ substituted for $PbCl_2$, on z-cut quartz.

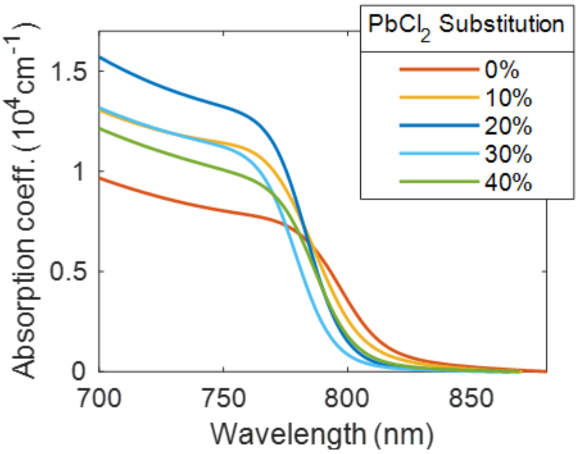


**Figure S10:** Absorption coefficient of vapor-deposited $FA_{1-y}Cs_yPb(I_{1-x}Cl_x)_3$ thin films grown with varying amounts of $PbI_2$ substituted for $PbCl_2$, on z-cut quartz.

### 5.2 Elliott Fits

The onset in the absorption coefficient spectrum is fitted using Elliott's model that accurately describes the absorption of a semiconductor near its band edge.

The theory expresses the energy-dependent absorption as:

$$\alpha(E) = \alpha_x(E) + \alpha_c(E)$$

The bound exciton contribution $\alpha_x$ has the form:

$$\alpha_x(E) = \frac{b_0}{E}\sum_{n=1}^{\infty}\frac{4\pi E_b^{3/2}}{n^3}\delta(E - [E_g - \frac{E_b}{n^2}])$$

Where $b_0$ is a constant of proportionality that incorporates the electric dipole transition matrix element between the valence and conduction band. $\alpha_x$ is formed of the weighted sum of contributions from the exciton states with positive integer quantum number *n* and energies $E_g - \frac{E_b}{n^2}$, where $E_g$ is the band gap energy and $E_b$ is the exciton binding energy.

Here, the contribution from the electron-hole continuum states $\alpha_c(E)$ has the form:

$$\alpha_c(E) = \frac{b_0}{E}\left[\frac{2\pi\sqrt{\frac{E_b}{E-E_g}}}{1-\exp\left(-2\pi\sqrt{\frac{E_b}{E-E_g}}\right)}\right] C_0^{-1}\mathrm{JDoS}(E)$$

Where the joint density of states is given by $\mathrm{JDoS}(E) = C_0\sqrt{E-E_g}$ for $E > E_g$ and 0 otherwise, and the joint density of states constant $C_0 = \frac{1}{(2\pi)^2}(\frac{2\mu}{\hbar^2})^{3/2} \times 2$, where μ is the reduced effective mass of the electron-hole system. The term in square brackets is the Coloumbic enhancement factor, which increases the absorption from the continuum states above the square-root form of the JDoS for a direct-gap semiconductor as a result of the Coloumbic attraction between the unbound electrons and holes.

The linear combination of the contributions from the excitonic and continuum states, *α(E)*, is then convolved with a broadening function comprising of a convolution of a normal distribution - representing the homogeneous broadening caused by electron-phonon coupling, and an asymmetric log-normal distribution - accounting for the inhomogeneous broadening caused by disorder and local fluctuations of the stoichiometry of the material. The Broadening is mathematically denoted by $g(E) = \aleph(0, \sigma_T^2) \circledast \ln(\aleph(u_s, \sigma_s^2))$.

For our series, the log-normal broadening parameters were globally fitted for all the films with different PbCl2 concentrations, while the band gap, binding energy and electron phonon coupling were freely fitted afterwards. Table S6 shows the fit parameters obtained for the Elliott fits to our series.

| **$PbCl_2$ Substitution** | **$E_g$ /eV** | **$E_b$ /meV** | **$\sigma_T$ /meV** | **$u_s$** | **$\sigma_s$** |
|---|---|---|---|---|---|
| 0% | 1.57366 | 9.66120 | 33.70808 | -6.93468 | 0.507652 |
| 10% | 1.58893 | 7.72837 | 33.15868 | -6.93468 | 0.507652 |
| 20% | 1.59800 | 7.81850 | 26.43606 | -6.93468 | 0.507652 |
| 30% | 1.60600 | 7.55779 | 26.80731 | -6.93468 | 0.507652 |
| 40% | 1.59359 | 8.60487 | 29.14552 | -6.93468 | 0.507652 |

**Table S6:** Fit parameters obtained from Elliott fits to the absorption edge of vapor-deposited $FA_{1-y}Cs_yPb(I_{1-x}Cl_x)_3$ thin films grown with varying amounts of $PbI_2$ substituted for $PbCl_2$, on z-cut quartz. $E_g$ is the bandgap, $E_b$ the exciton binding energy, $\sigma_T$ represents the homogeneous broadening caused by electron-phonon coupling, $u_s$ and $\sigma_s$ represent the inhomogenous broadening.

### 5.3 External Quantum Efficiency and Urbach Energy

The External Quantum Efficiency (EQE) was measured using a Fourier transform photocurrent spectrometer in the FTIR system described in section 5.1. The photocurrent was measured using a near-infrared source and a $CaF_2$ beamsplitter, using a Newport-calibrated reference silicon solar cell of known EQE value for calibration. Figure S11 shows the EQE for the 20% $PbCl_2$ device shown in the main text with the integrated current density, showing good agreement with the $J_{sc}$ measured under AM1.5 light. We also calculated the expected bandgap of the material from the EQE, and obtained values of 1.530 and 1.553 eV for the 0% and 30% $PbCl_2$ compositions, showing a similar shift to the values calculated from Elliott fits. Overall, Elliott fitting should provide the most accurate estimate of the bandgap [7].

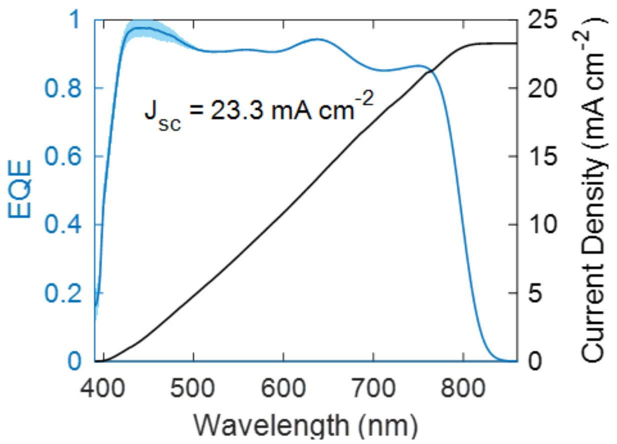


**Figure S11:** EQE (blue line) and the corresponding integrated current density (black line) at AM1.5 for a $FA_{1-y}Cs_yPb(I_{1-x}Cl_x)_3$ device grown with 20% of the $PbI_2$ substituted with $PbCl_2$. The light blue area represents the noise threshold for the measurement.

Urbach energy ($E_U$) measurements were taken on the same setup as the EQE, using a 700 nm low-pass filter, such that the absorption band edge could be measured at a much higher sensitivity. The single exponential $y = e^{\frac{(x-a)}{E_U}}$ was fitted to the data in the region near the band edge to obtain $E_U$. Three measurements were taken per sample to get an average, with the error reflecting the random error in the mean. These values are shown in Table S7.

| $PbCl_2$ Substitution | Urbach Energy Measurements /meV | | | $U_b$ Average /meV |
|---|---|---|---|---|
| 0% | 11.335 | 11.340 | 11.335 | 11.336(3) |
| 20% | 11.741 | 10.748 | 11.017 | 11.2(5) |

**Table S7:** Urbach energy ($E_U$) measured on 3 devices made from vapor-deposited $FA_{1-y}Cs_yPb(I_{1-x}Cl_x)_3$ with or without 20% of the $PbI_2$ substituted for $PbI_2$.

To the best of our knowledge, the $E_U$ value of 10.7 meV for the 20% $PbCl_2$ sample is the lowest value reported in the literature. To support this, we have listed reported literature values for the $E_U$ of various MHP materials as extracted from FTPS measurements in Table S8.

| Material | $E_u$ /meV | Reference |
|---|---|---|
| $MAPbI_3$ | 14.3 | Ledinsky et al., 2019 [8] |
| $MAPbI_3$ | 12.5 | Lohmann et al., 2020 [1] |
| $FA_{0.83}Cs_{0.17}Pb(I_{0.9}Br_{0.1})_3$ | 14.2 | Sutter-Fella et al., 2018 [9] |
| $MA_{0.6}FA_{0.4}PbI_3$ 2% Cs | 17.0 | Tang et al., 2017 [10] |
| $FAPbI_3$ 3% Cs, MDA | 26.9 | Kim et al., 2020 [11] |

**Table S8:** Urbach energy ($E_U$) values reported for different MHP compositions. The second row represents the previous lowest $E_U$ value we were able to achieve on our system for large grain $MAPbI_3$.

### 5.4 Photoluminescence Spectra

We performed photoluminescence (PL) measurements on thin films grown on z-cut quartz. These measurements were taken by illuminating the samples with a 470 nm picosecond pulsed diode laser (PicoQuant, LDH-D-C-470M) operated at 1 MHz, at a power of 30 µW. The PL emitted by the films was coupled into a grating spectrometer (Princeton Instruments, SP-2558), and measured using and iCCD (PI-MAX4, Princeton Instruments).

To obtain the peak wavelength position, a pseudo-Voigt distribution was fit to the data, following the same formula as used for the XRD fits. To account for slight asymmetry in the peaks, the pseudo-Voigt distribution was fit to the left edge of the data to get the most accurate peak position. Table S9 shows the extracted PL peak position, as used in Figure 2e.

| $PbCl_2$ Substitution | PL Peak Position /eV | Fit error /eV |
|---|---|---|
| 0% | 1.536 | 0.00017 |
| 10% | 1.545 | 0.00011 |
| 20% | 1.554 | 0.00009 |
| 30% | 1.565 | 0.00006 |
| 40% | 1.553 | 0.00009 |

**Table S9:** Photoluminescence (PL) peak positions obtained from the PL spectra shown in Figure 2d

### 5.5 Time-Resolved Photoluminescence

We performed time correlated single photon (TCSPC) experiments with a photon-counting detector (PDM series from MPD) and a PicoHarp300 TCSPC event timer. The samples were photoexcited by the same 470 nm picosecond pulsed diode laser (PicoQuant, LDH-D-C-470M) operated at repetition rate 1 MHz used for the steady-state PL. The emission was collected using a grating spectrometer (Princeton Instruments, SP-2558). For Figure 1b, the excitation fluence was around 20 nJ/cm$^2$ respectively. The TCSPC traces from Figure 1b were fit with stretched exponentials to obtain a mean lifetime, following the equations in the work of deQuilettes et al. [12]. Figure S12 shows the traces for the whole range of $PbCl_2$ substitution, along with the extracted average lifetimes which were shown in Figure 3a.

**(a)**

| PbCl$_2$ Substitution | Average Lifetime /ns | Fit error /ns |
|---|---|---|
| 0% | 5.6 | 0.000015 |
| 10% | 74.5 | 0.00009 |
| 20% | 104.7 | 0.0005 |
| 30% | 83.5 | 0.00013 |
| 40% | 128.5 | 0.0003 |

**(b)**

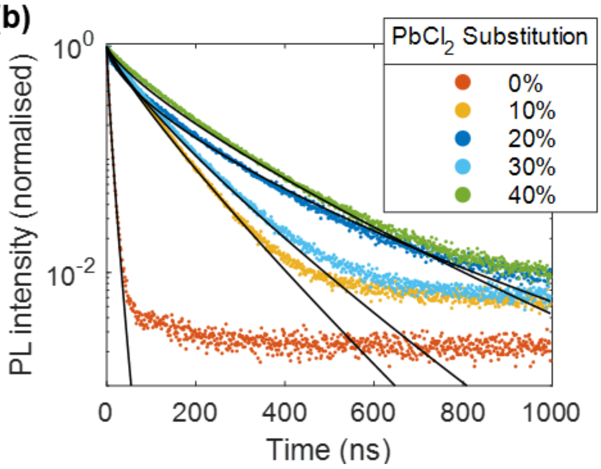


**Figure S12: (a)** Photoluminescence lifetimes of vapor-deposited $FA_{1-y}Cs_yPb(I_{1-x}Cl_x)_3$ thin films on z-cut quartz, grown with varying amounts of $PbI_2$ substituted for $PbCl_2$. The lifetimes were obtained from stretched exponential fits to the time-resolved photoluminescence (TRPL) traces, shown in **(b)**, after photoexcitation by a 1 MHz-pulsed 470 nm laser, at fluence 20 µJ/cm$^2$.

### 5.6 Optical Pump THz Probe Spectroscopy

Optical-Pump THz-Probe (OPTPS) experiments were performed as follows. A Spectra Physics Mai Tai–Empower–Spitfire Pro Ti:Sapphire regenerative amplifier provides 35 fs pulses centered at 800 nm at a repetition rate of 5 kHz. 400 nm photoexcitation is obtained by frequency doubling the fundamental laser output through a BBO crystal. THz probe pulses are generated by a spintronic emitter composed of 1.8 nm of $Co_{40}Fe_{40}B_{20}$ sandwiched between 2 nm of tungsten and 2 nm of platinum, all supported by a quartz substrate. Detection of the THz pulses was performed using electro-optic sampling in a ZnTe crystal (1 mm (110)-ZnTe). The samples studied were bare perovskite films deposited on z-cut quartz. The sample, THz emitter and THz detector were held under vacuum (<$10^{-2}$ mbar) during the measurements.

Figure S13 shows the OPTPS photoconductivity over pump delay for the samples studied here, with increasing content of $PbCl_2$.

The effective charge-carrier mobility was extracted from the amplitude of the OPTP signal immediately after photoexcitation (i.e. before charge-carrier recombination occurs). The sheet photoconductivity, ΔS, of a material with a thickness much shorter than the wavelength of the THz radiation can be expressed as

$$\Delta \mathrm{S} \;=\; -\epsilon_0 \mathrm{c}(\mathrm{n_a}+\mathrm{n_b})(\Delta T/T) \qquad \text{(S1)}$$

where $n_a$ and $n_b$ are the THz refractive indices of the materials interfacing the perovskite layer at the front and rear respectively. The quantity ΔT/T is the ratio of the photo-induced change in THz electric field to the transmitted THz electric field in the dark. The initial number of photo-excited charge carriers N is given by

$$\mathrm{N} \;=\; \varphi\frac{\mathrm{E}\lambda}{\mathrm{hc}}\left(1-\mathrm{T_{pump}}-\mathrm{R}_{pump}\right) \qquad \text{(S2)}$$

with E being incident pump pulse energy, λ the excitation wavelength, ϕ the ratio of free charges created per photon absorbed, and $R_{pump}$ and $T_{pump}$ being the reflected and transmitted fractions of the pump beam intensity. These two equations can be used to extract the charge-carrier mobility µ through

$$\mu = \frac{\Delta S A_{eff}}{Ne} \quad \text{(S3)}$$

where $A_{eff}$ is the effective area from the overlap of the pump and probe beams and $e$ is the elementary charge. Substituting Equations S1 and S2 into Equation S3 we obtain

$$\varphi\mu = -\frac{\epsilon_0 c(n_a+n_b)(A_{eff})}{Ne\lambda(1-R_{pump}-T_{pump})}\left(\frac{\Delta T}{T}\right) \quad \text{(S4)}$$

from which the effective charge-carrier mobility ϕμ may be determined based on the pump beam parameters and the initial measured ΔT/T of the sample. Here, μ is the charge-carrier mobility, and ϕ is the charge-to-photon branching ration which is assumed to be unity at room temperature.

The fluence dependence data for each sample was globally fitted to

$$\frac{dn}{dt} = -k_1 n - k_2 n^2 - k_3 n^3$$

Where n is the carrier density and $k_1$, $k_2$, and $k_3$ are the first, second, and third order recombination rate constants. Each film was modelled as a stack of 100 slices with initial carrier distribution in each slice calculated from the absorption profile of the 400 nm pump light. To account for the diffusion of charge-carriers, the population distribution across the film was allowed to vary as described previously by Crothers et al. [13]. The diffusion coefficient is calculated using the mobilities obtained experimentally. For simplicity, $k_3$ was fixed to $1 \times 10^{-30}$ $cm^6 s^{-1}$. The values for $k_1$ were adjusted to the decays at the lowest fluences, and $k_2$ was optimized in the fitting routine. The fits are shown alongside the OPTPS transients in Figure S13. Table S10 shows the values for the effective charge carrier mobilities, as well as the recombination rate constants obtained from the OPTPS measurements.

| **$PbCl_2$ Substitution** | **Mobility /$cm^2V^{-1}s^{-1}$** | **$k_2$ /$cm^3s^{-1}$** |
|---|---|---|
| 0% | 45(2) | $6.22 \times 10^{-10}$ |
| 10% | 51(4) | $10.2 \times 10^{-10}$ |
| 20% | 52(3) | $12.6 \times 10^{-10}$ |
| 30% | 57(3) | $20.5 \times 10^{-10}$ |
| 40% | 52(4) | $9.76 \times 10^{-10}$ |

**Table S10:** Charge carrier mobility and second order recombination rate obtained from OPTPs measurements

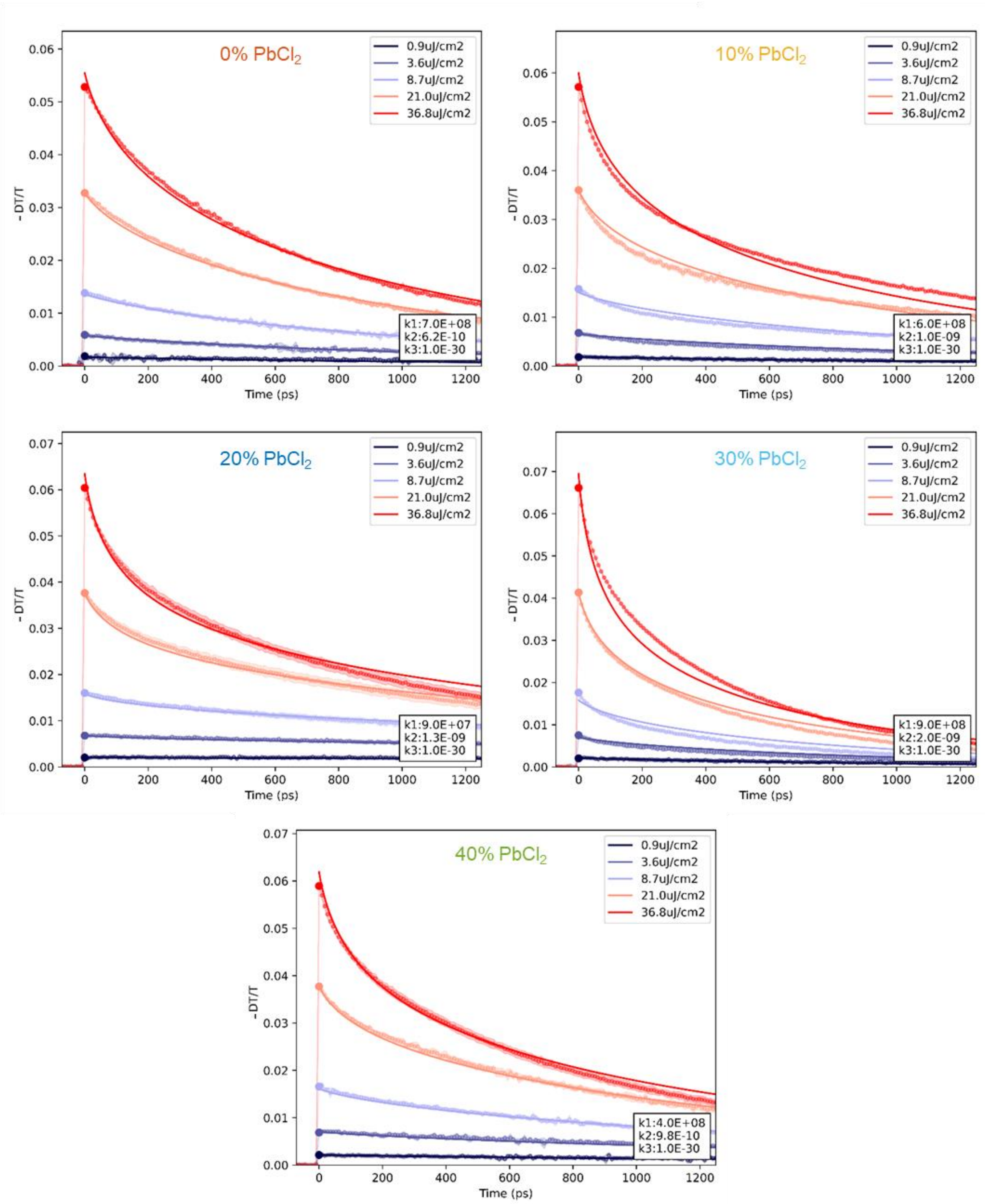


**Figure S13:** OPTPS photoconductivity transients obtained from vapor-deposited $FA_{1-y}Cs_yPb(I_{1-x}Cl_x)_3$ thin films on z-cut quartz, grown with varying amounts of $PbI_2$ substituted for $PbCl_2$. The thin solid lines represent the fits to the data.

### 5.7 Photoluminescence Quantum Yield

We performed photoluminescence quantum yield (PLQY) measurements following the procedure developed by de Mello et al. [14]. The samples were placed in an integrated sphere and photoexcited by a 532 nm laser the samples and the signal was collected by a QEPro spectrometer. The illumination intensity was 24 mWcm$^{-2}$ which corresponds to approximately 0.5 suns equivalent photon flux [15], therefore the PLQY values presented here are underestimates of their value at one-sun equivalent conditions. Table S11 shows the obtained PLQE values plotted in Figure 3b of the main text.

| **$PbCl_2$ Substitution** | **PLQY /%** |
|---|---|
| 0% | 0.02 |
| 10% | 0.08 |
| 20% | 0.16 |
| 30% | 0.17 |
| 40% | 0.3 |

**Table S11:** Photoluminescence quantum yield (PLQY) values obtained from exciting the vapor-deposited $FA_{1-y}Cs_yPb(I_{1-x}Cl_x)_3$ thin-films on z-cut quartz, with 24 mWcm$^{-2}$, 532 nm photoexcitation.

## 6. Cl Incorporation

### 6.1 Theory

Because our final perovskite layer consists of multiple components and phases, there are multiple ways for Cl to be used during film formation without ending up in the bulk perovskite. The raw $PbCl_2$ precursor can have the following reactions with the other precursors:

1) React with FAI to form $PbI_2$ and FACl, according to the following reaction

$$PbCl_2 + 2FAI \rightarrow PbI_2 + 2FACl$$

2) React with CsI to form $PbI_2$ and CsCl, according to the following reaction

$$PbCl_2 + 2CsI \rightarrow PbI_2 + 2CsCl$$

In general we can combine these first two equations by introducing a factor $\alpha$ representing the preference for CsCl formation over FACl, with $0 < \alpha < 1$:

$$PbCl_2 + 2\alpha CsI + 2(1-\alpha)FAI \rightarrow PbI_2 + 2\alpha CsCl + 2(1-\alpha)FACl \quad (1)$$

3) Remain unreacted

From our XRD results, we find the phases present in the final film to be $PbI_2$, $CsPbCl_3$, and some FA/Cs I/Cl mixed perovskite with general formula $FA_{1-y}Cs_yPb(I_{1-x}Cl_x)_3$, where $0 < x, y < 1$. As such, we can rule out large amounts of $PbCl_2$ remaining unreacted. Instead, the possible formation of $CsPbCl_3$ opens a new pathway for any CsCl formed, namely:

$$CsCl + \gamma PbCl_2 \rightarrow (1-\gamma)CsCl + \gamma CsPbCl_3 \quad (2)$$

Where $0 < \gamma < 1$ represents the percentage of formed CsCl that reacts with $PbCl_2$ to form $CsPbCl_3$ (i.e. if $\gamma = 0$ no $CsPbCl_3$ is formed, if $\gamma = 1$ only $CsPbCl_3$ is formed. If we multiply equation (2) by $2\alpha$, we can substitute into equation (1), yielding:

$$(1 + 2\gamma\alpha)PbCl_2 + 2\alpha CsI \; + 2(1 - \alpha)FAI \\ \rightarrow PbI_2 + 2\alpha(1 - \gamma)CsCl + 2(1 - \alpha)FACl + 2\alpha\gamma CsPbCl_3 \qquad (3)$$

We can now write down a general equation for the ratio of products based on the factors $a, b, \alpha, \gamma$, which define the relative amount of CsI to FAI, the relative amount of $PbI_2$ to $PbCl_2$, the relative amount of CsCl formed to FACl formed, the relative amount of $CsPbCl_3$ formed for each CsCl. First, we start from the precursors evaporated:

$$(1 - a)FAI + aCsI + (1 - b)PbI_2 + bPbCl_2 \\ = (1 - a)FAI + aCsI + (1 - b)PbI_2 + \frac{b}{1 + 2\gamma\alpha}(1 + 2\gamma\alpha)PbCl_2 \\ + \frac{b}{1 + 2\gamma\alpha}[2\alpha CsI + 2(1 - \alpha)FAI] - \frac{b}{1 + 2\gamma\alpha}[2\alpha CsI + 2(1 - \alpha)FAI] \\ \rightarrow [1 - a - 2f(1 - \alpha)]FAI + (a - 2f\alpha)CsI + (1 - b + f)PbI_2 + 2f\alpha(1 - \gamma)CsCl \\ + 2f(1 - \alpha)FACl + 2f\alpha\gamma CsPbCl_3 \qquad (4)$$

Here, we have added the terms in the orange box (which sum to 0) to obtain a multiple $f = \frac{b}{1+2\gamma\alpha}$ of equation (3) with the terms from the grey box. Note that this equation is only valid for sufficient amounts of CsI, i.e. $a \geq 2f\alpha$. This represents the moment when all CsI has reacted to form either CsCl or $CsPbCl_3$. These cases will be discussed later on. From equation (4), we can see that Cl could enter the bulk perovskite through CsCl or FACl. If we assume that FACl is volatile enough to leave the film during deposition or annealing, but CsCl is able to integrate the bulk perovskite, equation (4) can be rearranged into the following products:

$$FA_{[1-a-2f(1-\alpha)]}Cs_{(a-2f\alpha\gamma)}Pb_{[1-b+f(2\alpha-1)]}I_{[3-2b-4f(1-\alpha)]}Cl_{2f\alpha(1-\gamma)} + 2f\alpha\gamma CsPbCl_3 \\ + f(1 - \alpha)PbI_2 + 2f(1 - \alpha)FACl \qquad (5)$$

From this, we can deduce the fraction of Cs to FA and Cl to I in the bulk perovskite, denoted by $y$ and $x$ respectively in a composition of the form $FA_{1-y}Cs_yPb(I_{1-x}Cl_x)_3$:

$$y = \frac{a - 2f\alpha\gamma}{1 - b + f(2\alpha - 1)}$$

$$x = \frac{2f\alpha(1 - \gamma)}{3[1 - b + f(2\alpha - 1)]}$$

These values can then be used to determine the expected bandgap and lattice parameter of the resulting perovskite.

As mentioned earlier, this relationship only holds true while there is enough CsI in the precursors to allow both the desired CsCl and $CsPbCl_3$ fraction to form. Should this no longer be the case, when $a < 2f\alpha$, there are two scenarios available:

1) Fix the current fraction of CsCl and $CsPbCl_3$, meaning all additional $PbCl_2$ reacts with FAI only
2) Allow the $CsPbCl_3$ to continue to grow, now removing CsCl from the bulk perovskite, until all Cs is in the $CsPbCl_3$ phase, at which point all additional $PbCl_2$ reacts with FAI only.

Our data shows an initial increase in the bandgap and lattice constant, with the trend reaching a maximum around 20-30% $PbCl_2$ substitution, followed by a reversal in the trend. Because the only process that could reduce the bandgap and increase the lattice size in our system is the removal of Cs and Cl from the bulk perovskite, we expect the second process to be dominant in our system. With these assumptions we can do simple modelling of the expected lattice parameter and bandgap as a function of $PbCl_2$ substitution. Figure S14 shows 4 different scenarios based on the fraction of $CsPbCl_3$ formed. The model calculates the Cs and Cl fraction in the bulk perovskite according to the relationships derived above. The bandgap is then calculated assuming linear shifts from one material to the next. Here, we use values of 1.54 eV for $FAPbI_3$, previously reported for vapor-deposited films using Elliott fits [16], 1.81 eV for $CsPbI_3$, previously reported at room temperature from Elliot fits [17], and 3.0 eV for $CsPbCl_3$ [18]. To summarize, we obtain the following expression for the bandgap as a function of Cs and Cl substitution (denoted by $0 < y, x < 1$ respectively) of $FAPbI_3$.

$$E_g = 1.54 + 0.27y + 1.19x$$

We note that our coefficient of *y*, 0.27, is very similar to that previously reported by Li et al. of 0.28 [19], even though we derive ours from Elliott fits where theirs comes from Tauc plots. In reality, there may be band-bending occurring, especially for the poorly understood I-Cl substitution, since it is observed for I-Br substitution [20]. Band-bending for I-Cl substitution in the same direction as I-Br substitution would result in an underestimate of I-Cl substitution for low *x* values, so we expect the numerical values obtained to be approximate. Nevertheless, the trends obtained should still reflect the ones observed in our material. The resulting bandgap was calculated for a variety of α and γ values across a range of $PbCl_2$ substitution. For ease of understanding, the curves were grouped according to the fraction of $CsPbCl_3$ grown per 3 Cl atoms in the starting products. The resulting bulk perovskite can be seen to vary wildly depending on the formation of $CsPbCl_3$, FACl, and the latter's volatility. Comparing to the trends found for our samples in Figure 2b and 2e, we see that we are likely forming a medium amount of $CsPbCl_3$ per 3 Cl atoms in the starting products, between 0.4 and 0.8 $CsPbCl_3$ per 3 Cl, depending on how much FACl is formed versus CsCl. Importantly, we can see that, in order to obtain the trends we observe, we need some amount of Cl to incorporate into the lattice and increase the bandgap, such that not all Cl we detect in the

films can come from $CsPbCl_3$. In the following sub-sections we will explore what we can deduce about the formation of FACl and CsCl.

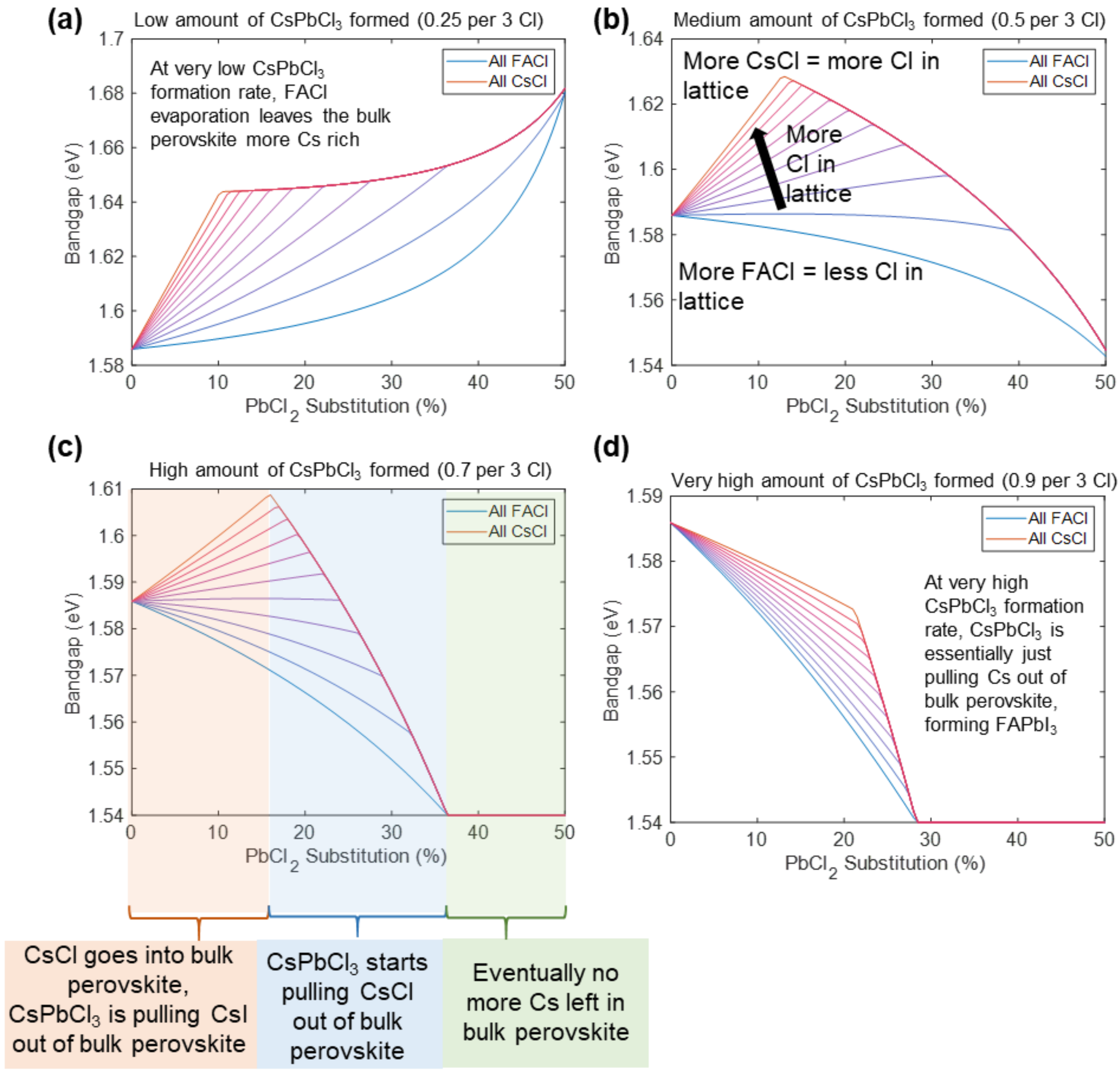


**Figure S14:** Modelling of expected bandgap of $FA_{1-y}Cs_yPb(I_{1-x}Cl_x)_3$, depending on $PbCl_2$ substitution of $PbI_2$, $CsPbCl_3$ formation, and relative preference of $PbCl_2$ to form FACl or CsCl. The $CsPbCl_3$ formation rate is defined as the fraction of $CsPbCl_3$ formed per 3 Cl added, i.e. at 1 all Cl goes to form $CsPbCl_3$. The $CsPbCl_3$ formation rates shown here are **(a)** 0.25 **(b)** 0.5 **(c)** 0.7 **(d)** 0.9. Within each individual graph, the blue line represents the scenario where, other than $CsPbCl_3$, only FACl is formed. The red line represents the opposite scenario, where only CsCl is formed, while the gradient of lines in between represents mixed scenarios with more or less CsCl, as depicted in **(b)**. Effectively, the "all FACl" scenario depicted by the blue line represents no Cl substituting into the bulk perovskite, while the "all CsCl" scenario represents all Cl not forming $CsPbCl_3$ substituting into the bulk perovskite.

### 6.2 Evidence for Cl in the Final Films

To determine how much Cl is present in the final films, we performed XPS and EDX measurements on the series of films going from 0% to 40% $PbCl_2$ substitution. The high-resolution spectra of the Cl 2*p* region measured in XPS are shown in Figure 2c. Figure S15a shows the calculated I:Pb:Cl ratios from the XPS measurements, normalized to the amount of Pb, and Figure S15b graphically shows the calculated Cl/(I+Cl) ratio as a function of $PbCl_2$ substitution. We can see that we are clearly detecting substantial amounts of Cl in the final films, of the order of 1-10%, with the amount of Cl detected increasing as more $PbCl_2$ is added. However, the numerical value of Cl detected is only about half of what was originally evaporated. Lab-based XPS measurements, like the ones carried out in this work, probe depths of up to 10 nm therefore to ensure the detection of Cl was not limited to the surface of the films we also performed EDX measurements on another set of films. Figure S16a shows the calculated Cl/(I+Cl) ratio as a function of $PbCl_2$ measured from films used for devices (ITO/PTAA/Perovskite/C60/BCP), while Figure S16b shows EDX measurements for a bare film of the champion 20% $PbCl_2$ composition before and after annealing. We observe a similar trend to the XPS measurements, with the Cl detected increasing with added $PbCl_2$, but also more Cl in general, up to 20% for the film with 40% $PbCl_2$ substitution. We also find only a small change in Cl content before and after annealing, with the final annealed 20% $PbCl_2$ thin film showing 11.5% Cl, very close to the amount of Cl evaporated, 13.3% (Figure S16b). Overall, our results show that most of the Cl sublimated ends up in the final film, with only a few percent lost during deposition or annealing.

**(a)**

| $PbCl_2$ Substitution | XPS Relative Atomic Composition (normalised) | | | Measured Cl/(I+Cl) /% |
|---|---|---|---|---|
| | Pb | I | Cl | |
| 0% | 1.00(2) | 2.65(3) | 0 | 0 |
| 10% | 1.00(3) | 2.47(3) | 0.05(2) | 2.2(0.8) |
| 20% | 1.00(3) | 2.39(4) | 0.09(3) | 3.6(1.2) |
| 30% | 1.00(3) | 2.37(3) | 0.11(4) | 4.2(1.4) |
| 40% | 1.00(3) | 2.20(3) | 0.31(4) | 12.2(1.6) |

**(b)**

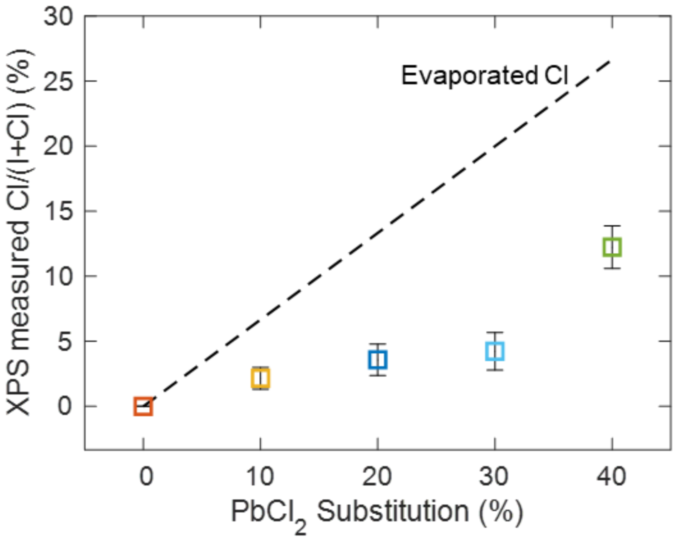


**Figure S15: (a)** Relative atomic composition measured using X-ray photoemission spectroscopy (XPS), normalized to Pb content. **(b)** Graphical representation of the relative Cl percentage measured in XPS, using the values from the last column of (a). The dashed line represents the Cl/(I+Cl) ratio of the precursors evaporated.

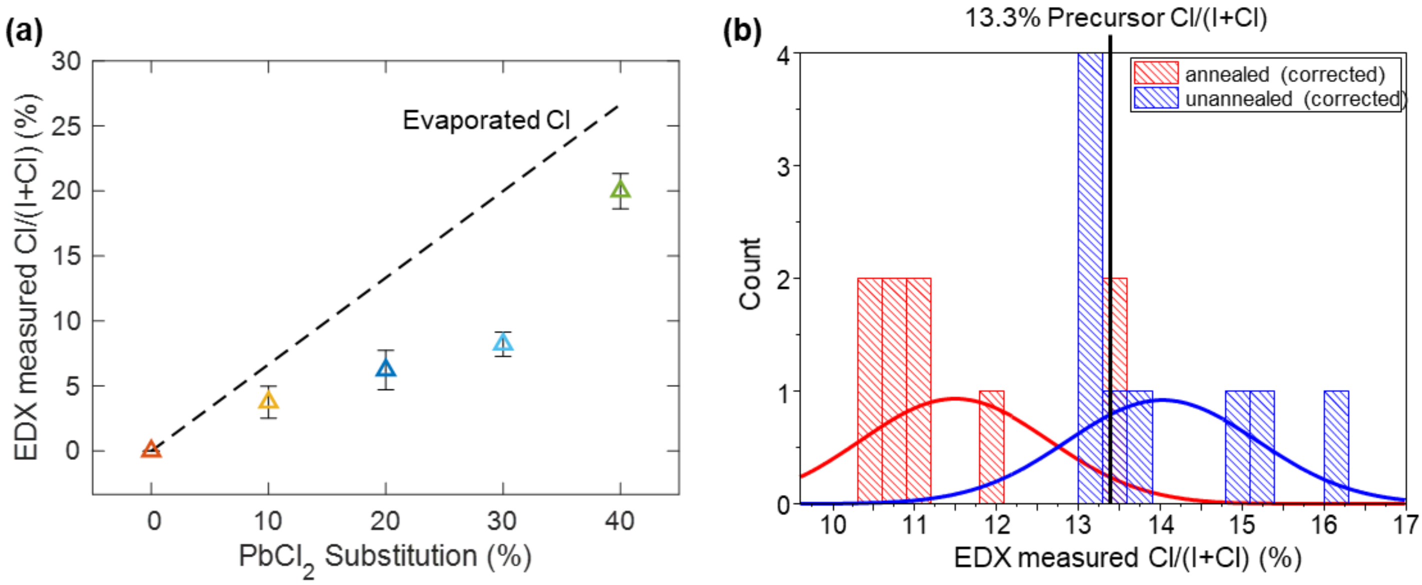


**Figure S16: (a)** Graphical representation of the relative Cl content measured using energy dispersive x-ray spectroscopy (EDX), from the $FA_{1-y}Cs_yPb(I_{1-x}Cl_x)_3$ devices grown with varying amounts of $PbI_2$ substituted for $PbCl_2$. The corresponding numerical values are found in Table S5. The dashed line represents the Cl/(I+Cl) ratio of the precursors evaporated. **(b)** Histogram of the relative Cl content measured using EDX for the annealed and unannalead film with 13% Cl at the precursor stage. The black line shows the Cl content present at the precursor stage.

### 6.3 Evidence for FACl Loss

The relative amount of FACl formed during growth and how much of it is re-sublimated has important consequences on the amount of Cl and I present in the final films. We believe that, if formed, FACl is likely volatile enough to escape the film during deposition or annealing, based on the behavior observed for MACl in $MAPbI_3$ grown from MAI and $PbCl_2$. Specifically, early solution processed perovskite recipes [21], and indeed the first thermal evaporation perovskite PV publication [22] employed $PbCl_2$ as the sole Pb source, and relied on volatilization of excess MACl to result in a post annealed film comprising predominantly $MAPbI_3$, with only a small amount of Cl substitution. Figure S17 shows the neat Cl perovskite phase XRD peak for films before and after annealing, and reveals that, while after annealing the position matches that of $CsPbCl_3$ in all cases, the position shifts to lower angles before annealing for higher $PbCl_2$ substitution. This reduction in XRD peak position implies an increase in lattice size of the neat Cl perovskite phase, consistent with the increased presence of FA in this phase. As such, this shows this is evidence that, for higher amounts of $PbCl_2$, we are indeed forming FACl during deposition, that not all FACl is lost through the high-vacuum during deposition, but that it is all lost through the annealing process.

Equation (5) shows that $PbI_2$ formation is associated with loss of FACl. We find that the XRD $PbI_2$ peak intensity drops for low amounts of $PbCl_2$ substitution but increases again from 30% $PbCl_2$ onwards (Figure S2). This is in good agreement with our evidence from the neat Cl phase XRD peak, specifically that there is little FACl loss for $PbCl_2$ substitution below 30%, but noticeable FACl formation and loss for $PbCl_2$ substitution over 30%. Finally, we expect the thickness of the resulting films to decrease as more FACl is lost, which we can observe in the SEM cross-sections (Figure S8). It is clear that, while the films from 0% to 30% $PbCl_2$ substitution generally have the expected thickness of 500 nm, the 40% $PbCl_2$ film is noticeably thinner than the others, consistent with increased loss of material.

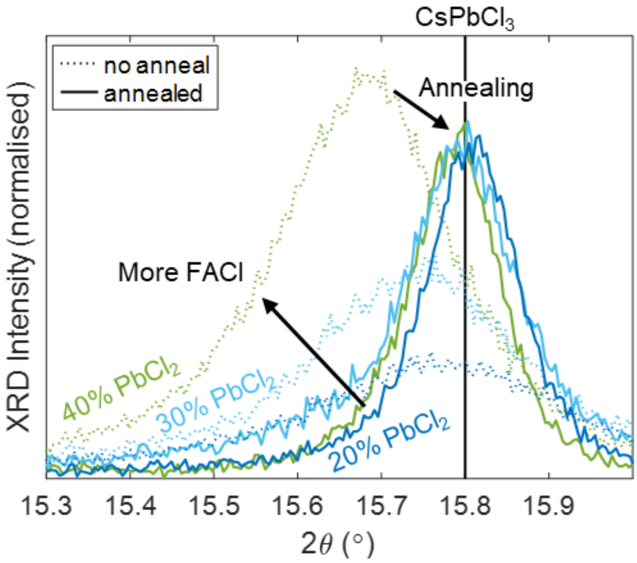


**Figure S17:** X-ray diffraction traces of vapor-deposited $FA_{1-y}Cs_yPb(I_{1-x}Cl_x)_3$ films on ITO/PTAA, grown with 20-40% of the $PbI_2$ substituted for $PbCl_2$. The dashed lines represent films before annealing, the solid lines represent films after a 30 minute anneal at 135 °C. The solid black line represents the position of the $CsPbCl_3$ (100) peak.

The most striking observation is that, while the 30% $PbCl_2$ and especially the 40% $PbCl_2$ films show evidence for FACl formation and loss, this is not the case for the 10% and 20% $PbCl_2$ films. From equation (5), we can deduce that this is likely related to preferential formation of CsCl over FACl i.e., larger values of α. Since the amount of Cs in the film is limited, FACl formation will dominate once all CsI has reacted with $PbCl_2$, and this is exactly what we observe.

### 6.4 Importance of $CsPbCl_3$ Formation

In general, we found the films grown with small amounts of Cl, before the formation of $CsPbCl_3$ to be less crystallographically stable than films with $CsPbCl_3$. Notably, the 10% $PbCl_2$ film, which does not show a $CsPbCl_3$ peak in XRD, is very unstable before annealing, in contrast to the other films (Figure S4). We were able to replicate this instability in films where the $PbCl_2$ was introduced separately from the bulk perovskite deposition, as a thin interlayer between the ITO/PTAA and the perovskite. Specifically, we deposited 3 nm and 9 nm of $PbCl_2$ followed by 500 nm of $FA_{0.83}Cs_{0.17}PbI_3$, grown without $PbI_2$ excess to account for the additional $PbCl_2$. We found that, for 3 nm of $PbCl_2$, the films are very unstable, quickly turning into δ-phase $CsPbI_3$ (Figure S18b), in contrast with the films grown on 9 nm of $PbCl_2$, which showed improved photovoltaic performance (Figure S19), accompanied by the appearance of a $CsPbCl_3$ peak (Figure S18a). As such, we speculate that the formation of at least some $CsPbCl_3$ is necessary to stabilize a mixed I-Cl perovskite phase.

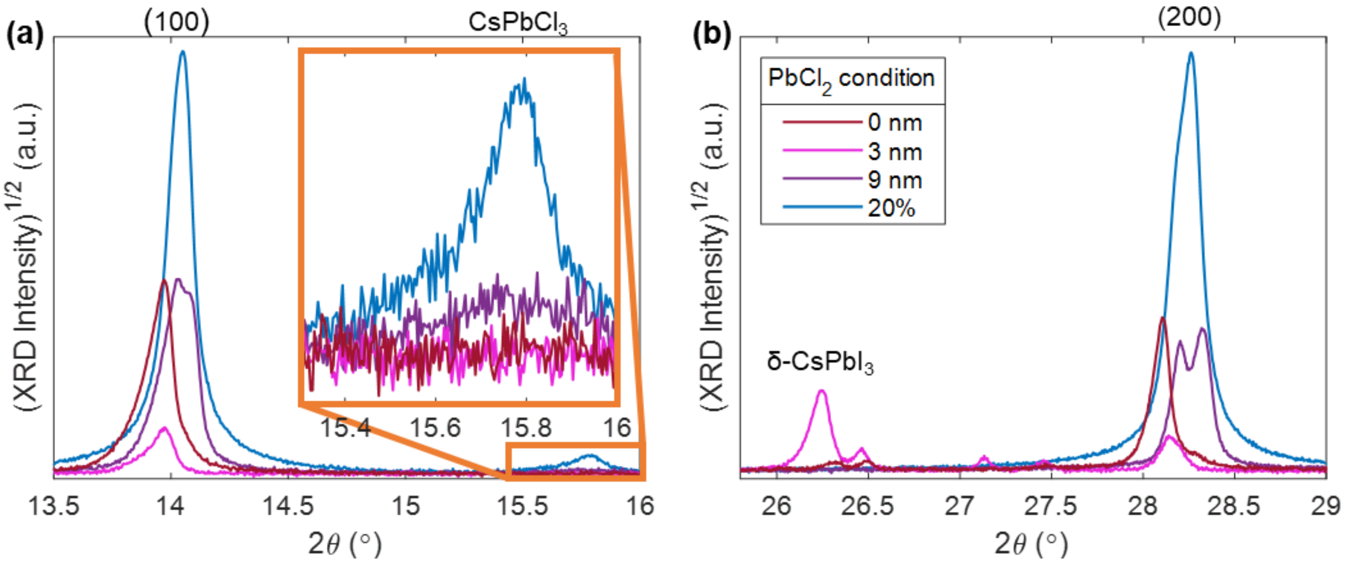


**Figure S18:** X-ray diffraction (XRD) patterns of vapor-deposited $FA_{1-y}Cs_yPb(I_{1-x}Cl_x)_3$ full devices after testing, grown either on top of a $PbCl_2$ interlayer or with 20% $PbI_2$ substituted for $PbCl_2$, as denoted by the legend. The y-axis shows the square root of the measured intensity to be able to show both the large perovskite peak and the much smaller $CsPbCl_3$ or δ-phase $CsPbI_3$ peak. **(a)** shows the XRD pattern around the bulk perovskite (100) peak, with the inset showing the same pattern zoomed in on the $CsPbCl_3$ (100) peak. **(b)** shows the XRD pattern around the (200) bulk perovskite peak

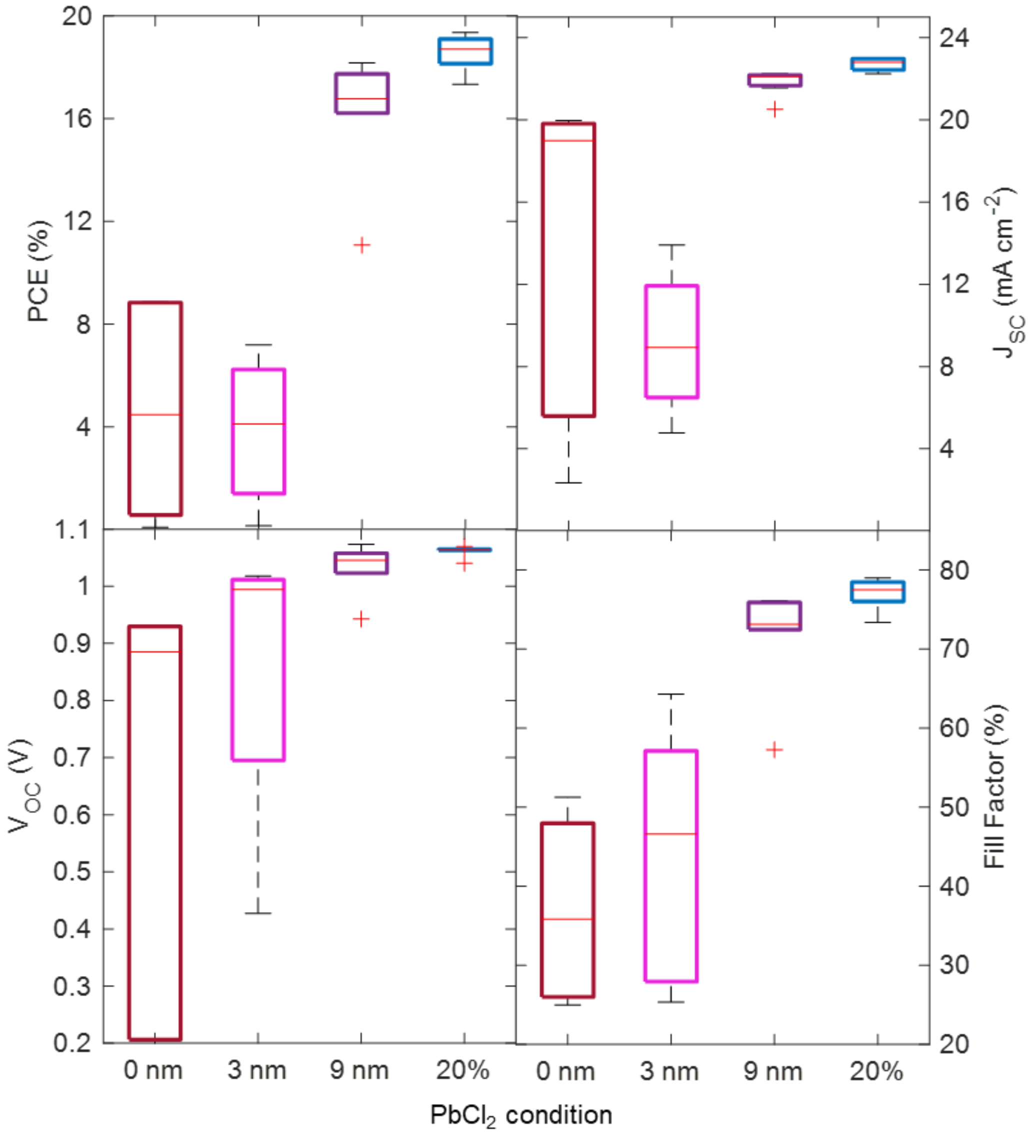


**Figure S19:** J-V characteristics of the reverse scan for co-evaporated $FA_{0.83}Cs_{0.17}PbI_3$ devices, grown with either a 0, 3, 9 nm $PbCl_2$ interlayer before the perovskite, or with 20% of the $PbI_2$ substituted for $PbCl_2$.

## 7. Extra J-V Data

Table S12 shows the numerical values of the J-V characteristics of the reverse scans for the $FA_{0.83}Cs_{0.17}Pb(I_{1-x}Cl_x)_3$ devices shown in the main text. Figure S20 shows the reverse scan for the champion device of each Cl composition investigated in the main text. We measured both 0.25 $cm^2$ and 1 $cm^2$ devices, and found very similar performance in general. Figure S21 shows a comparison of the 1 $cm^2$ device and the average J-V of the 0.25 $cm^2$ device for the 20% $PbCl_2$ composition. Next, as mentioned in the main text, some of the devices made with 30% $PbCl_2$ ended up phase unstable under sunlight illumination, turning yellow-colored, resulting in significantly reduced performance. Figure S22 shows the PCE of these discolored devices as compared to the normal ones shown in the main text, as well as an image of a film showing the very noticeable phase-transition of these pixels. Finally, Figure S23 shows an example of the batch-to-batch reproducibility of our system, showing a comparison of two different solar cell batches of the control $FA_{0.83}Cs_{0.17}PbI_3$ composition, showing average PCE within 2%.

| Precursor Cl/(I+Cl) /% | PCE /% | $J_{SC}$ /mAcm$^{-2}$ | $V_{OC}$ /V | FF /% |
|---|---|---|---|---|
| 0 | 16.4 (*15.53*) | 20.9 (*20.05*) | 1.03 (*1.038*) | 76.3 (*74.54*) |
| 7 | 18.0 (*17.04*) | 23.0 (*22.86*) | 1.01 (*1.000*) | 77.7 (*74.51*) |
| 13 | 19.3 (*18.56*) | 23.0 (*22.70*) | 1.06 (*1.061*) | 79.0 (*77.05*) |
| 20 | 15.9 (*14.56*) | 21.0 (*20.06*) | 1.06 (*1.046*) | 71.6 (*69.35*) |
| 27 | 14.0 (*11.44*) | 20.7 (*19.68*) | 1.05 (*1.030*) | 64.4 (*55.85*) |

**Table S12:** J-V characteristics of the reverse scans of evaporated $FA_{0.83}Cs_{0.17}Pb(I_{1-x}Cl_x)_3$ devices with various amounts of Cl. The table shows the champion device data with the average device data in italics, in parentheses.

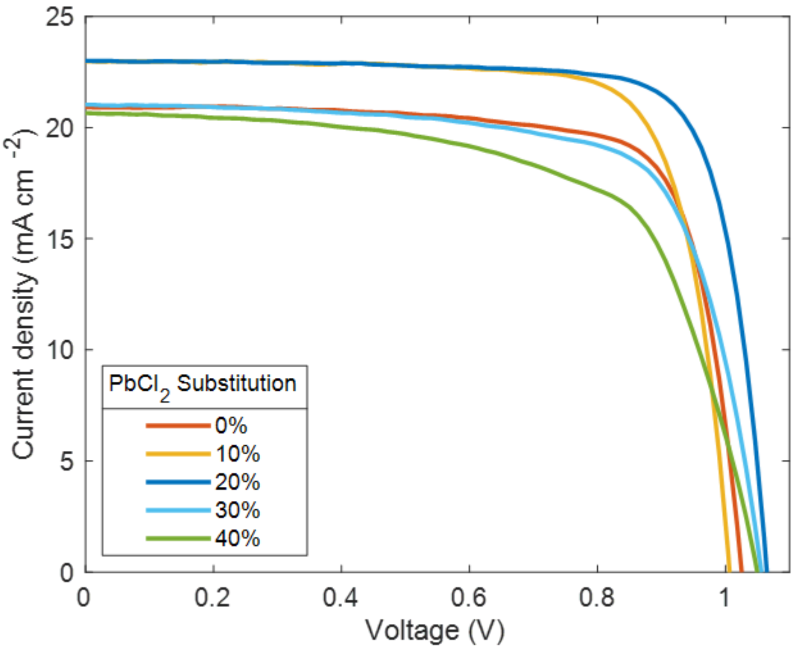


**Figure S20:** Reverse J-V scans for the champion device of the champion $FA_{0.83}Cs_{0.17}PbI_3$ devices, grown with varying amounts of $PbI_2$ substituted with $PbCl_2$, as denoted in the legend.

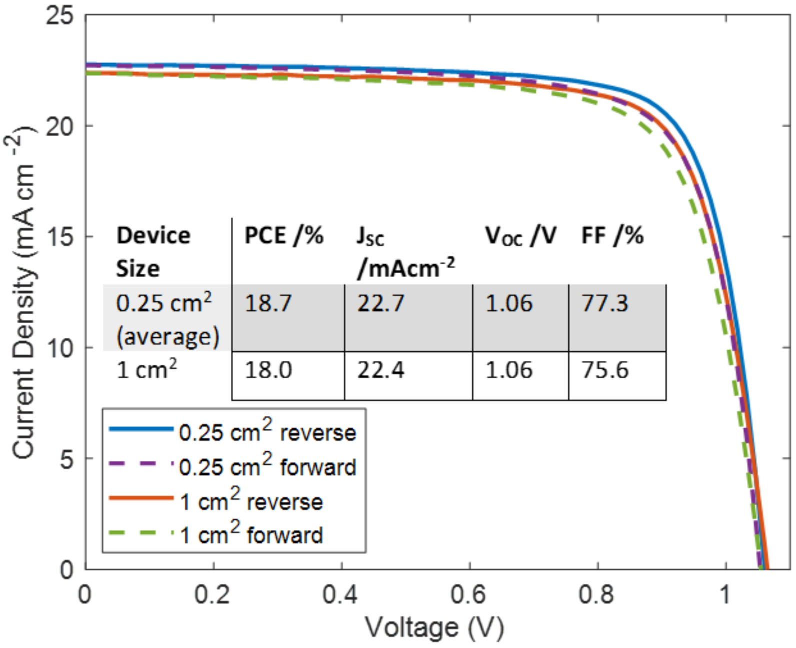


**Figure S21:** J-V scans for vapor-deposited $FA_{1-y}Cs_yPb(I_{1-x}Cl_x)_3$ devices, grown with 20% of the $PbI_2$ substituted with $PbCl_2$ at the precursor stage, according to device size. The J-V curve for the 0.25 $cm^2$ devices was calculated by averaging the J-V curves of all 0.25 $cm^2$ devices. The inset table shows the J-V figures of merit for the reverse scans shown in the figure.

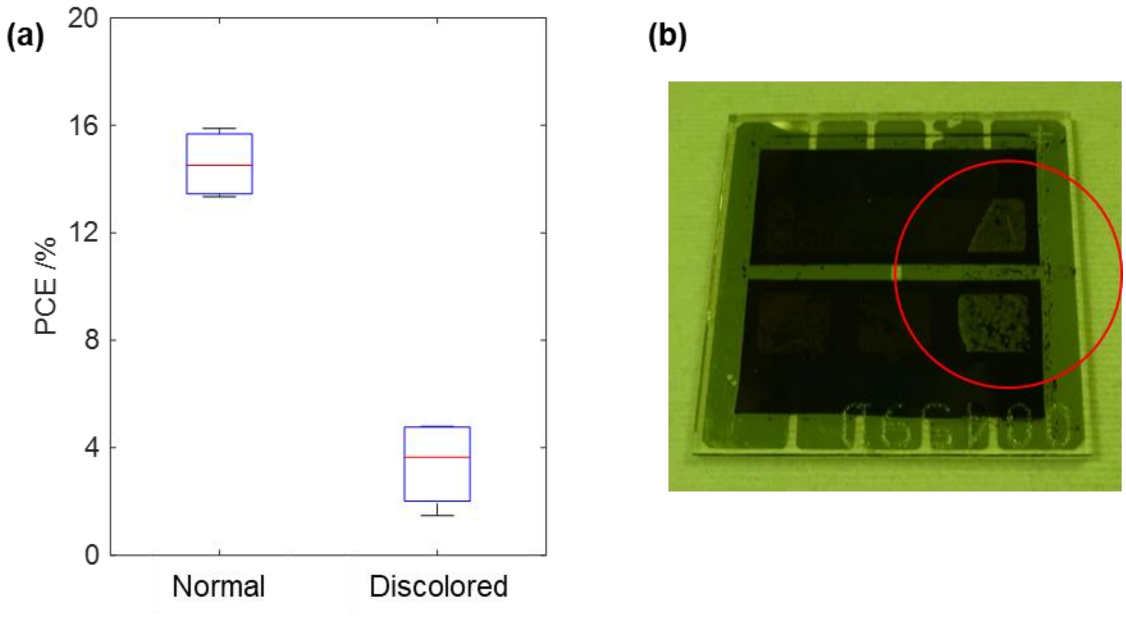


**Figure S22: (a)** J-V characteristics for vapor-deposited $FA_{1-y}Cs_yPb(I_{1-x}Cl_x)_3$ devices, grown with 30% of the $PbI_2$ substituted with $PbCl_2$ at the precursor stage, according to whether the devices showed normal behavior, or discoloration after illumination. **(b)** A photograph of one of the solar cells tested, showing two discolored devices circled in red.

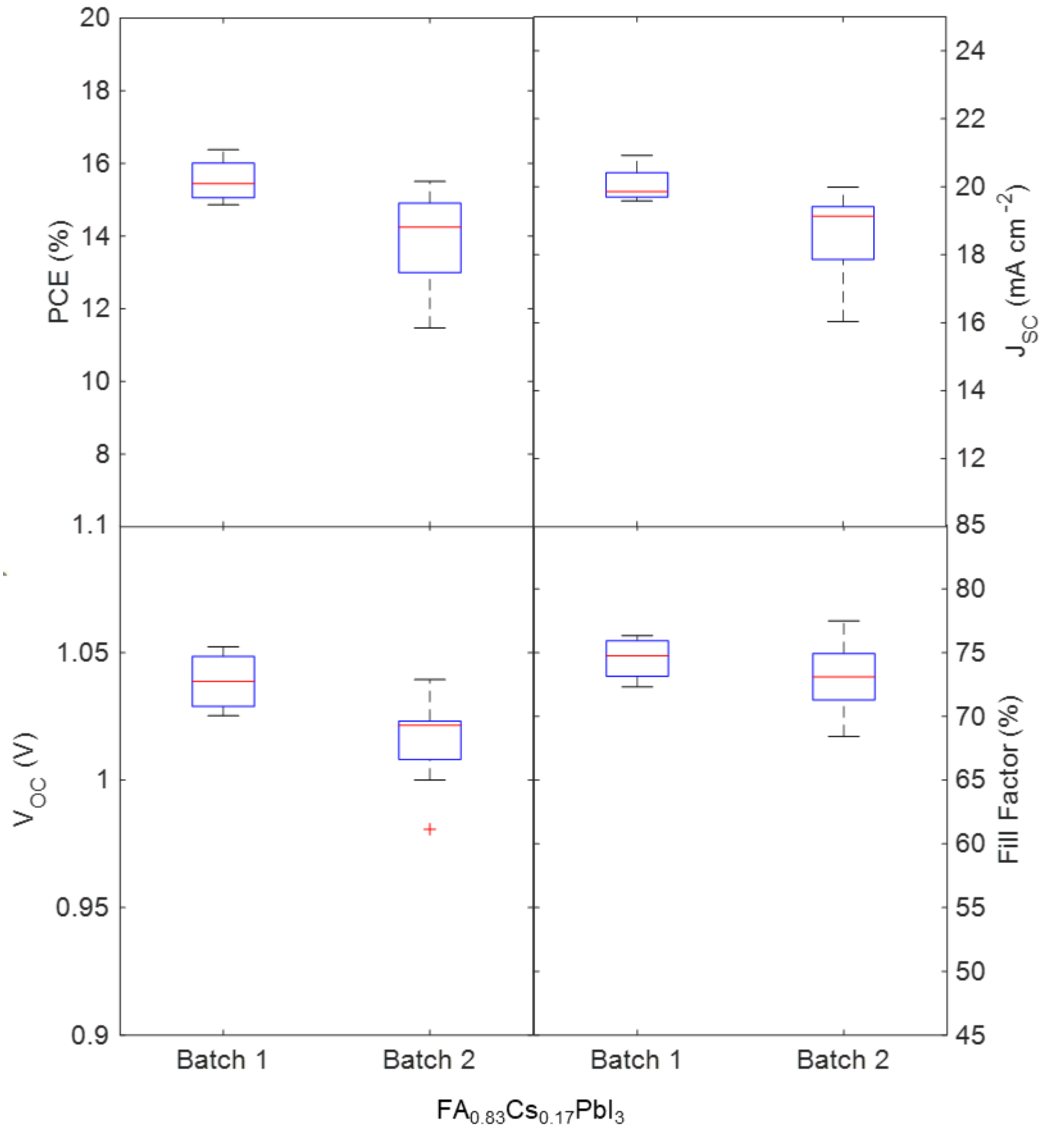


**Figure S23:** J-V characteristics of the reverse scan for co-evaporated $FA_{0.83}Cs_{0.17}PbI_3$ devices from two different deposition batches.

## 8. Effect of annealing

Figures S4 and S5 show the XRD patterns of our films before and after annealing at 135 °C for 30 mins, and show that for all compositions except the 10% $PbCl_2$ film, the MHP is already in the desired phase before annealing, and stable in ambient air. In the case of the 10% $PbCl_2$ film, it is only after annealing that the film is in the desired phase, and stable in air. The annealing process mainly increases the height of the XRD perovskite peaks, as shown in Figure S24a. We observe a similar behavior for the optical absorption coefficient, which already shows the desired absorption onset prior to annealing, with the latter slightly sharpening the onset (Figure S24b). Moreover, we found that annealing is required to ensure stability of the films under AM1.5 and applied bias, as solar cells made from unannealed films turned into the yellow δ-phase under testing conditions. Finally, as discussed in Section 6.3 of the SI, the annealing also shifts the pure Cl peak phase to 15.8% for compositions with $PbCl_2 \geq 30\%$, indicating loss of FACl.

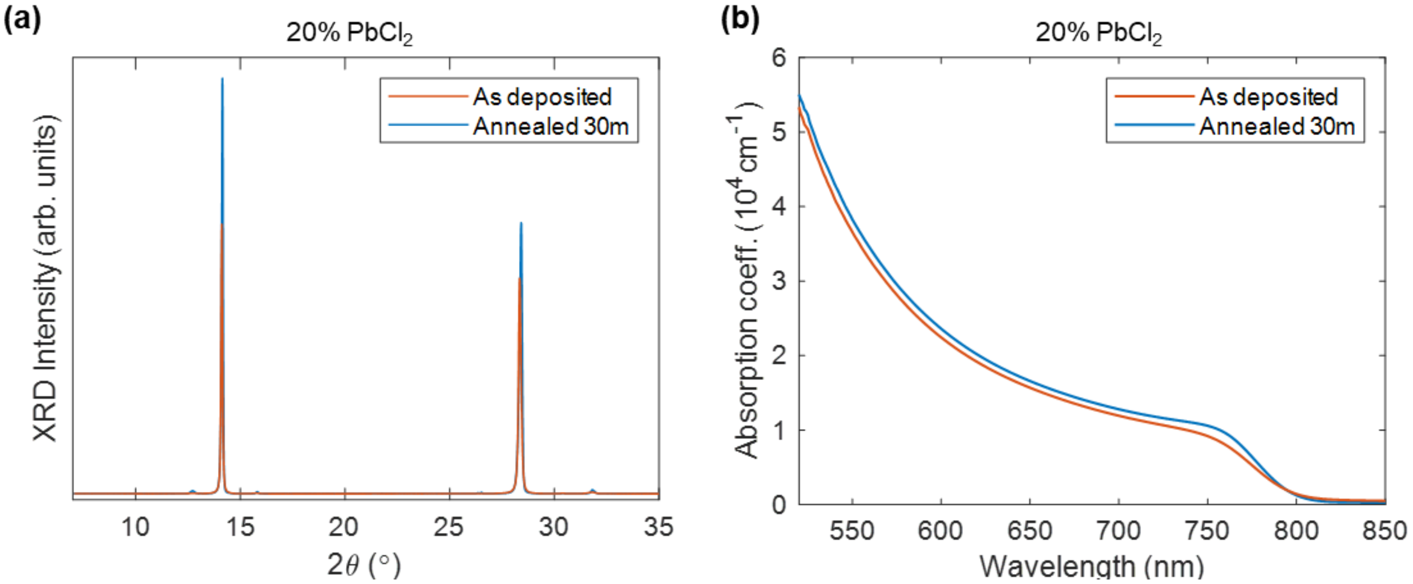


**Figure S24: (a)** X-ray diffraction (XRD) pattern of vapor-deposited $FA_{1-y}Cs_yPb(I_{1-x}Cl_x)_3$ thin films grown with 20% of the $PbI_2$ substituted for $PbCl_2$, on ITO/PTAA, as deposited (red) or after 30 minutes annealing at 135 °C. **(b)** Absorption coefficient of similar vapor-deposited films on z-cut quartz, as deposited (red) or after 30 minutes annealing at 135 °C.

## References


[1] K. B. Lohmann, J. B. Patel, M. U. Rothmann, C. Q. Xia, R. D. J. Oliver, L. M. Herz, H. J. Snaith and M. B. Johnston, "Control over Crystal Size in Vapor Deposited Metal-Halide Perovskite Films," *ACS Energy Lett.,* vol. 5, p. 710, 2020.

[2] P. Thompson, D. E. Cox and J. B. Hastings, "Rietveld refinement of Debye-Scherrer synchrotron X-ray data from Al2O3," *J. Appl. Cryst.,* vol. 20, p. 79, 1987.

[3] A. Ferrari and F. Giorgio, "Crystal Structure of the Iodides of Divalent Metals," *REND LINCEI-MAT APPL,* vol. 10, p. 522, 1929.

[4] N. Nadaud, N. Lequeux, M. Nanot, J. Jové and T. Roisnel, "Structural Studies of Tin-Doped Indium Oxide (ITO) and In4Sn3O12," *J. Solid State Chem.,* vol. 135, p. 140, 1998.

[5] M. T. Weller, O. J. Weber, J. M. Frost and A. Walsh, "Cubic Perovskite Structure of Black Formamidinium Lead Iodide, α-[HC(NH2)2]PbI3, at 298 K," *J. Phys. Chem. Lett.,* vol. 6, p. 3209, 2015.

[6] D. M. Trots and S. V. Myagkota, "High-Temperature Structural Evolution of Caesium and Rubidium Triiodoplumbates," *J. Phys. Chem. Solids,* vol. 69, p. 2520, 2008.

[7] C. L. Davies, J. Borchert, C. Q. Xia, R. L. Milot, H. Kraus, M. B. Johnston and L. M. Herz, "Impact of the Organic Cation on the Optoelectronic Properties of Formamidinium Lead Triiodide," *J. Phys. Chem. Lett.,* vol. 9, pp. 4502-4511, 2018.

[8] M. Ledinsky, T. Schönfeldová, J. Holovský, E. Aydin, Z. Hájková, L. Landová, N. Neyková, A. Fejfar and S. D. Wolf, "Temperature Dependence of the Urbach Energy in Lead Iodide Perovskites," *J. Phys. Chem. Lett.,* vol. 10, pp. 1368-1373, 2019.

[9] C. M. Sutter-Fella, Q. P. Ngo, N. Cefarin, K. L. Gardner, N. Tamura, C. V. Stan, W. S. Drisdell, A. Javey, F. M. Toma and I. D. Sharp, "Cation-Dependent Light-Induced Halide Demixing in Hybrid Organic–Inorganic Perovskites," *Nano Lett.,* vol. 18, pp. 3473-3480, 2018.

[10] S. Tang, Y. Deng, X. Zheng, Y. Bai, Y. Fang, Q. Dong, H. Wei and J. Huang, "Composition Engineering in Doctor-Blading of Perovskite Solar Cells," *Adv. Energy Mater.,* vol. 7, p. 1700302, 2017.

[11] G. Kim, H. Min, K. S. Lee, D. Y. Lee, S. M. Yoon and S. I. Seok, "Impact of strain relaxation on performance of a-formamidinium lead iodide perovskite solar cells," *Science,* vol. 370, pp. 108-112, 2020.

[12] D. W. deQuilettes, S. M. Vorpahl, S. D. Stranks, H. Nagaoka, G. E. Eperon, M. E. Ziffer, H. J. Snaith and D. S. Ginger, "Impact of microstructure on local carrier lifetime in perovskite solar cells," *Science,* vol. 348, p. 683, 2015.

[13] T. W. Crothers, R. L. Milot, J. B. Patel, E. S. Parrott, J. Schlipf, P. Müller-Buschbaum, M. B. Johnston and L. M. Herz, "Photon Reabsorption Masks Intrinsic Bimolecular Charge-Carrier Recombination in CH3NH3PbI3 Perovskite," *Nano Lett.,* vol. 17, p. 5782, 2017.

[14] D. J. C. d. Mello, D. H. F. Wittmann and P. R. H. Friend, "An improved experimental determination of external photoluminescence quantum efficiency," *Adv. Mater.,* vol. 9, p. 230, 2004.

[15] T. Kirchartz, J. A. Márquez, M. Stolterfoht and T. Unold, "Photoluminescence-Based Characterization of Halide Perovskites for Photovoltaics," *Adv. Energy Mater.,* vol. 10, p. 1904134, 2020.

[16] A. D. Wright, G. Volonakis, J. Borchert, C. L. Davies, F. Giustino, M. B. Johnston and L. M. Herz, "Intrinsic quantum confinement in formamidinium lead triiodide perovskite," *Nature Materials,* vol. 19, p. 1201, 2020.

[17] R. J. Sutton, M. R. Filip, A. A. Haghighirad, N. Sakai, B. Wenger, F. Giustino and H. J. Snaith, "Cubic or Orthorhombic? Revealing the Crystal Structure of Metastable Black-Phase CsPbI3 by Theory and Experiment," *ACS Energy Lett.,* vol. 3, pp. 1787-1794, 2018.

[18] K. Heidrich, W. Schafer, M. Schreiber, J. Sochtig, G. Trendel and J. Treusch, "Electronic structure, photoemission spectra, and vacuum-ultraviolet optical spectra of CsPbCl3 and CsPbBr3," *Physical Review B,* vol. 24, pp. 5642-5649, 1981.

[19] Z. Li, M. Yang, J.-S. Park, S.-H. Wei, J. J. Berry and K. Zhu, "Stabilizing Perovskite Structures by Tuning Tolerance Factor: Formation of Formamidinium and Cesium Lead Iodide Solid-State Alloys," *Chem. Mater.,* vol. 28, p. 284, 2016.

[20] J. H. Noh, S. H. Im, J. H. Heo, T. N. Mandal and S. I. Seok, "Chemical Management for Colorful, Efficient, and Stable Inorganic–Organic Hybrid Nanostructured Solar Cells," *Nano Lett.,* vol. 13, p. 1764, 2013.

[21] M. M. Lee, J. Teuscher, T. Miyasaka, T. N. Murakami and H. J. Snaith, "Efficient Hybrid Solar Cells Based on Meso-Superstructured Organometal Halide Perovskites," *Science,* vol. 338, p. 643, 2012.

[22] M. Liu, M. B. Johnston and H. J. Snaith, "Efficient planar heterojunction perovskite solar cells by vapour deposition," *Nature,* vol. 501, p. 395, 2013.
